# A comparison of fMRI presurgical mapping techniques with intraoperative brain mapping-based validation

**DOI:** 10.1162/imag_a_00280

**Published:** 2024-08-29

**Authors:** Ahmed M. Radwan, Louise Emsell, Kristof Vansteelandt, Evy Cleeren, Ronald Peeters, Steven De Vleeschouwer, Tom Theys, Patrick Dupont, Stefan Sunaert

**Affiliations:** Translational MRI, Department of Imaging and Pathology, KU Leuven, Leuven, Belgium; Department of Neurosciences, Leuven Brain Institute (LBI), KU Leuven, Leuven, Belgium; Neuropsychiatry, Department of Neurosciences, KU Leuven, Leuven, Belgium; Department of Geriatric Psychiatry, University Psychiatric Center (UPC), KU Leuven, Leuven, Belgium; Department of Neurology, UZ Leuven, Leuven, Belgium; Department of Neurosurgery, UZ Leuven, Leuven, Belgium; Department of Radiology, UZ Leuven, Leuven, Belgium; Research Group Experimental Neurosurgery and Neuroanatomy, Department of Neurosciences, KU Leuven, Leuven, Belgium; Department of Neurosciences, Laboratory for Cognitive Neurology, KU Leuven, Leuven, Belgium

**Keywords:** functional MRI, presurgical mapping, resting-state fMRI, task-based fMRI, multiecho fMRI, intraoperative brain mapping, direct electrical stimulation

## Abstract

Resting-state functional MRI (rsfMRI) could enable preoperative risk assessment and intraoperative guidance for patients who cannot undergo task-based fMRI (tbfMRI). To ascertain rsfMRI’s applicability, we investigated differences in accuracy between tbfMRI with a voxel size of 1.8 x 1.8 x 3.2 mm³ and rsfMRI acquired with single-echo (sTE) with a voxel size of 2 x 2 x 2.2 mm³ and multiecho (mTE) with a voxel size of 3 x 3 x 3 mm³ using intraoperative mapping with direct electrical stimulations (DES) as the ground truth. Functional sensory-motor mapping results of hands and feet were spatially compared relative to positive (pDES, functional effect) and negative (nDES, no functional effect) coordinates in 16 preoperative patients. A general linear model analysis was used for tbfMRI, and seed-based analysis (SBA) for rsfMRI. Minimum Euclidean distances between fMRI and DES were calculated and compared between fMRI methods. Receiver-operating characteristic (ROC) curves were used to compare accuracy and determine distance cutoffs for fMRI agreement with DES, and binary agreement rates were compared at different cutoffs. Two-part mixed-effects linear models were used to compare fMRI methods while accounting for unequal intersubject DES repetition. Only minor differences were found between fMRI methods in unthresholded distances (mean differences ~2 mm). ROCs and binary agreement measures showed comparable accuracy for tbfMRI and sTE-rsfMRI at 2 mm, but mildly worse for sTE-rsfMRI at 3 mm and mTE-rsfMRI. However, differences in relative accuracy between sTE-rsfMRI and mTE-rsfMRI were minor when the same distance cutoff was applied to all methods. This was also reflected in comparing binary agreement rates and confirmed by the two-part linear models, which showed no significant differences between fMRI methods and a significant effect of DES response. A similar accuracy for SBA rsfMRI functional sensory-motor mapping compared with tbfMRI for the hands and feet indicates that rsfMRI may be suitable for presurgical mapping. The differences in relative accuracy between sTE-rsfMRI and mTE-rsfMRI warrant further investigation in a larger sample.

## 1 Introduction

Blood oxygen-level dependent (BOLD) task-based functional magnetic resonance imaging (tbfMRI) is routinely used in clinical practice for presurgical brain mapping. TbfMRI is typically used for sensory-motor and language mapping and for estimating hemispheric language dominance (Lehéricy et al., 2000;Stippich et al., 2007;Sunaert, 2006;Wengenroth et al., 2011). Functional brain mapping with tbfMRI has been previously validated against intraoperative direct electrical stimulation (DES) (Fandino et al., 1999;Jack et al., 1994;Lehéricy et al., 2000;Xie et al., 2008), which is the gold-standard method for functional brain mapping. The use of fMRI for presurgical brain mapping was also recently shown to be associated with decreased morbidity and mortality in brain tumor patients undergoing surgical resection (Vysotski et al., 2018). BOLD tbfMRI can be used for mapping lower-order brain functions such as vision, hearing, sensation, and movement, as well as higher-order brain functions, such as language, memory, and attention. Each function is typically mapped using a 3–5 minutes scan, which means that mapping multiple functional domains with tbfMRI would be exceedingly time consuming. While this may be acceptable in neuroscientific settings, where volunteers might tolerate longer scanning times and can be sufficiently trained for task performance, it is not usually tolerable for clinical patients whose task performance can degrade with longer scan times due to increasing discomfort, distraction, or fatigue (Bennett & Miller, 2013;Hausman et al., 2022;Morrison et al., 2016). In addition, some patients may not be able to perform a task at all either due to cognitive impairment, lack of understanding, language, or educational difficulties, very young or old age, or requiring sedation. All these issues are further compounded by the wide variation of functional mapping results due to specific task differences (Niskanen et al., 2012;Unadkat et al., 2019), and task-related head motion (Kochiyama et al., 2005).

In contrast, resting-state fMRI (rsfMRI) (Biswal et al., 1995), which measures the spontaneous fluctuation of BOLD signal and its correlation between different brain regions as functional connectivity, requires no task performance and a single scan of 5–7 minutes can typically be used to map multiple lower- and higher-order resting-state networks (RSNs). RsfMRI can also be acquired with sedation, or while watching a video that can soothe very young patients who cannot hold still without an audiovisual stimulus (Pur et al., 2021). This makes it an attractive alternative to tbfMRI and assessing its applicability in a clinical setting is the focus of considerable debate and research. Multiple studies have shown good concordance between rsfMRI compared with intraoperative mapping for different functional domains (Cochereau et al., 2016;Lu et al., 2017;Qiu et al., 2014;Rosazza et al., 2014). However, it suffers from a number of limitations which may reduce its adoption in clinical neuroradiology. The absence of a task makes the interpretation of functional mapping based on rsfMRI functional connectivity more challenging. For example, a tbfMRI scan with a finger-tapping task shows the so-called functionally-eloquent areas of hand representation in the primary sensory-motor cortices, whereas rsfMRI shows the resting-state network (RSN) of sensory-motor (SM) function without directly differentiating eloquent and non-eloquent parts. There is also a lack of consensus on analysis methods, and a relative lack of clinical guidelines (Fox & Greicius, 2010;Gonzalez-Castillo et al., 2021;Lee et al., 2016;O’Connor & Zeffiro, 2019). Furthermore, while there are multiple data analysis tools for expert neuroscientists (Friston et al., 2007;Cox, 1996;Jenkinson et al., 2012), there are only a few user-friendly analysis packages (Hsu et al., 2018;Leuthardt et al., 2018;Whitfield-Gabrieli & Nieto-Castanon, 2012;Zacà et al., 2018) available for clinical research, and none provided by any major commercial health-tech vendors for rsfMRI. In contrast there is a wide variety of such clinically approved tools for tbfMRI data analysis.

Both task-based and rsfMRI data are subject to the confounding effect of head motion, EPI distortion, and susceptibility effects. One recent development, multiecho time (mTE)-fMRI, aims to reduce these effects at the acquisition stage, and, therefore, has potential utility in the clinic.

MTE-fMRI acquires T2*-weighted images with at least three echo times (TE), thus increasing signal-to-noise ratio (SNR) and providing a relative resilience to mild motion artifacts. While motion artifacts can cause B0 inhomogeneities, as well as susceptibility distortion—and dropouts—by motion interactions, leading to changes in both S0 and R2* with some TE dependence. In most brain regions, except areas close to air–tissue interfaces, such as the orbitofrontal cortex, the effects of motion on T2* are minor compared with S0-related signal changes, which are largely independent of TE (Andersson et al., 2001;Hutton et al., 2002;Van et al., 2023). In contrast, true neuronal BOLD signal changes drive only R2* changes and are largely TE dependent, with a TE of about 30 msec at 3 Tesla, showing maximal sensitivity to neuronal BOLD signal changes. Additionally, mTE-fMRI can partially recover BOLD signals in regions typically obscured by strong EPI distortion and susceptibility artifacts (Kundu et al., 2012).

To the best of our knowledge, the relative accuracy of tbfMRI, sTE-rsfMRI, and mTE-rsfMRI has not been validated against intraoperative functional mapping with DES in the same patient sample.

## 2 Aim of the Study

In this study, we compared mapping results for the sensory-motor functions of the hands and feet from tbfMRI, sTE-rsfMRI at 2 mm and 3 mm voxel size, and mTE-rsfMRI to intraoperative functional mapping with DES using fully automated methods. We quantitatively compared the three fMRI methods in a sample of 16 neurosurgical patients who underwent preoperative MRI-based and intraoperative DES-based functional mapping to investigate the suitability of the rsfMRI results for presurgical planning. We hypothesized that there would be no significant differences in the proximity of functional maps and DES coordinates among the four fMRI methods.

## 3 Methodology

### 3.1 Research questions

This study attempted to provide an in-depth understanding of the interplay between DES response, fMRI methods, fMRI tasks, and their influence on proximity and agreement between fMRI activity and DES coordinates. To do so, we investigated the following research questions:

First**(RQ1)**, “How do the different fMRI methods compare in terms of raw distance measures between fMRI maps and DES coordinates?” then**(RQ2)**“How do fMRI methods compare on receiver-operating characteristic (ROC) curves?” Third,**(RQ3)**“How do fMRI methods compare in terms of binary agreement and disagreement with DES at different distance cutoffs?” and lastly,**(RQ4)**“Are there significant differences between fMRI methods when accounting for the intersubject repeated measures?”

### 3.2 Participants

We recruited 79 surgery-naïve patients who were referred to our department for presurgical fMRI and DTI between January 2019 and January 2021, 16 patients underwent presurgical tbfMRI and intraoperative mapping with matching cortical DES for the hands and/or feet. All participating patients, and/or their legal guardians, signed written informed consent before participation, in accordance with the Declaration of Helsinki. Local ethics committee approval was acquired (UZ/KU Leuven, Leuven, Belgium, study number S61759). Participating patients were excluded if they had undergone previous therapeutic brain surgery, had brain implants, for example, deep brain stimulation electrodes, ventriculoperitoneal shunts, etc., or had absolute contraindications to MRI scanning. Summarized demographics and pathology information can be found inTable 1and further detailed inSupplementary Table 1.

**Table 1.** Summarized patient demographics and pathology information.

Age and Gender	Lesion		
	Type	Laterality	Cerebral lobar distribution
Age range = 9–73 years 10 males 6 females Median age = 39.5 years Interquartile range = 28 years	14 neoplasms: 13 gliomas (HGG) 1 meningioma 2 focal cortical dysplasia	7 right sided 9 left sided	2 frontoparietal 1 frontotemporal 8 frontal 2 parietal 1 parieto-occipital1 Temporofrontoparietal 1 Frontotemporoparieto-occipital (multifocal lesion)

HGG = High-grade glioma (World Health Organization grade > 2).

### 3.3 MRI acquisition

Two 3-Tesla whole-body MRI scanners were used for multimodal presurgical scanning (Ingenia - Elition, and Achieva DStream, Philips Medical Systems, Best, The Netherlands). Both MRI scanners were equipped with 32-channel phased-array receiver head coils. The acquisition parameters for the 3D T1-weighted images, T2-weighted images, and T2 fluid attenuated inversion recovery (FLAIR) images were previously described (Radwan et al., 2021).Table 2lists the acquisition parameters of BOLD tbfMRI (single-echo), sTE-rsfMRI, and mTE-rsfMRI data. The first patient (PT001) differed from the rest for sTE-rsfMRI, which was acquired with TR = 950 ms, multiband = 8, voxel size = 2 x 2 x 2 mm, while PT002 had 250 volumes for rsfMRI acquisitions.

**Table 2.** MRI acquisition parameters.

Acquisition parameters/fMRI methods	tbfMRI	sTE-rsfMRI	mTE-rsfMRI
Acquisition plane—Pulse sequence	Axial—2D gradient-echo EPI		
TR/TE ms: FA°	1500/33: 80	900/33: 65	1150/8 - 33 - 58: 75
Voxel size mm ^3^	1.8 x 1.8 x 3.2	2 x 2 x 2.2	3 x 3 x 3
Acquisition matrix	112 x 112 x 44	112 x 112 x 66	80 x 80 x 48
In-plane SENSE/Multiband SENSE	2.1/2	1.2/6	1.9/4
Pixel BW	2162	2044	2253
PE—Fat shift directions	AP – P		
Number of volumes	120-160	500	400

MRI = magnetic resonance imaging, tbfMRI = task-based functional MRI, sTE-rsfMRI = single-echo resting-state fMRI, mTE-rsfMRI = multiecho rsfMRI, EPI = echo planar imaging, TR = repetition time, TE = echo time, ms = milliseconds, FA = flip angle, SENSE = SENSitivity encoding in plane parallel imaging acceleration, BW = bandwidth, PE = phase encoding, AP = anteroposterior, P = posterior, DES = direct electrical stimulation.

### 3.4 Functional MRI

A symmetrical block design consisting of 3–4 pairs of 30-second blocks of task performance versus neutral observation as a control condition was used for tbfMRI scanning. All tasks were explained and exercised before scanning. Sensory-motor tasks involved finger-tapping or fist-clenching for the hands, and movement of the toes. Patients were instructed to engage in mind-wandering for rsfMRI while a black screen was shown. This was substituted with watching a video, movie, or cartoon of the patients’ choosing if they were unable to remain still without a visual stimulus. Visual stimuli for tbfMRI were synchronized with the scanner and displayed using Presentation (Neurobehavioral Systems, NC, USA) via a projector system to a flat in-bore plastic projector screen or via an MRI-compatible display. In both cases, the visual stimuli, for example, start, move, and stop, rest, were visible to the patient via a mirror mounted on top of the head coil and MRI-compatible corrective glasses were used if needed. Task performance was monitored in real-time on the scanner console by evaluating the inline results using the Philips iViewBOLD application. TbfMRI and sTE-rsfMRI were acquired for all patients, while mTE-rsfMRI was not acquired for two patients (PT006 and PT010).

### 3.5 Intraoperative brain mapping

Wake-up neurosurgery and cortical intraoperative DES were performed for the 16 patients included in this study. Intraoperative frameless neuronavigation (Curve, BrainLab, Munich, Germany) was employed in all cases. Cortical DES used the OSIRIS neurostimulator (Inomed Medizintechnik GmbH, Germany), and a bipolar fork stimulator with 5 mm interelectrode spacing (Inomed) for cortical mapping. Stimulation parameters followed the protocols described byDuffau et al. (1999)(60 Hz) in anesthetized patients and the low-frequency protocol described byZangaladze et al. (2008)(5 Hz) in awake patients.Table 3lists the DES tests done, the number of resulting positive and negative DES coordinates, effects of positive DES responses, and fMRI tasks of interest for each patient. Baseline cortical DES mapping was performed immediately after the dura was opened and the patient awakened, and before resection. Test sites were selected based on visible anatomical features in the surgical area and on coregistered MRI scans. Sites subjected to DES were indicated with sterile square markers, saved on the neuronavigation system, and tested repeatedly during resection. This study included only cortical coordinates for comparison with fMRI results.

**Table 3.** DES intraoperative brain mapping protocol details.

Coded patient names	Awake surgery	MEP/SSEP	Stimulation range in mA	Cortical/subcortical threshold
PT001	yes	no	4–20 mA	20 mA / -
PT002	no	yes	4–14 mA	6 mA / 4 mA
PT003	yes	no	4–6 mA	4 mA
PT004	yes	no	4–20 mA	- / 5 mA
PT005	yes	no	4–20 mA	16 mA / 5 mA
PT006	yes	no	4–20 mA	16 mA / 10 mA
PT007	yes	no	4–20 mA	20 mA / 2 mA
PT008	yes	no	2–20 mA	8 mA / -
PT009	yes	no	4–20 mA	-
PT010	yes	no	4–20 mA	-
PT011	yes	no	4–20 mA	12 mA / -
PT012	yes	no	4–20 mA	12 mA / 5 mA
PT013	yes	no	4–20 mA	20 mA / 5 mA
PT014	yes	no	4–20 mA	20 mA / 10 mA
PT015	yes	no	4–20 mA	- / 10 mA
PT016	yes	no	4–15 mA	4 mA / 4 mA

DES = direct electrical stimulation, MEP/SSEP = motor and somatosensory evoked potentials, mA = milliampere.

### 3.6 Data analysis

**Fig. 1.**
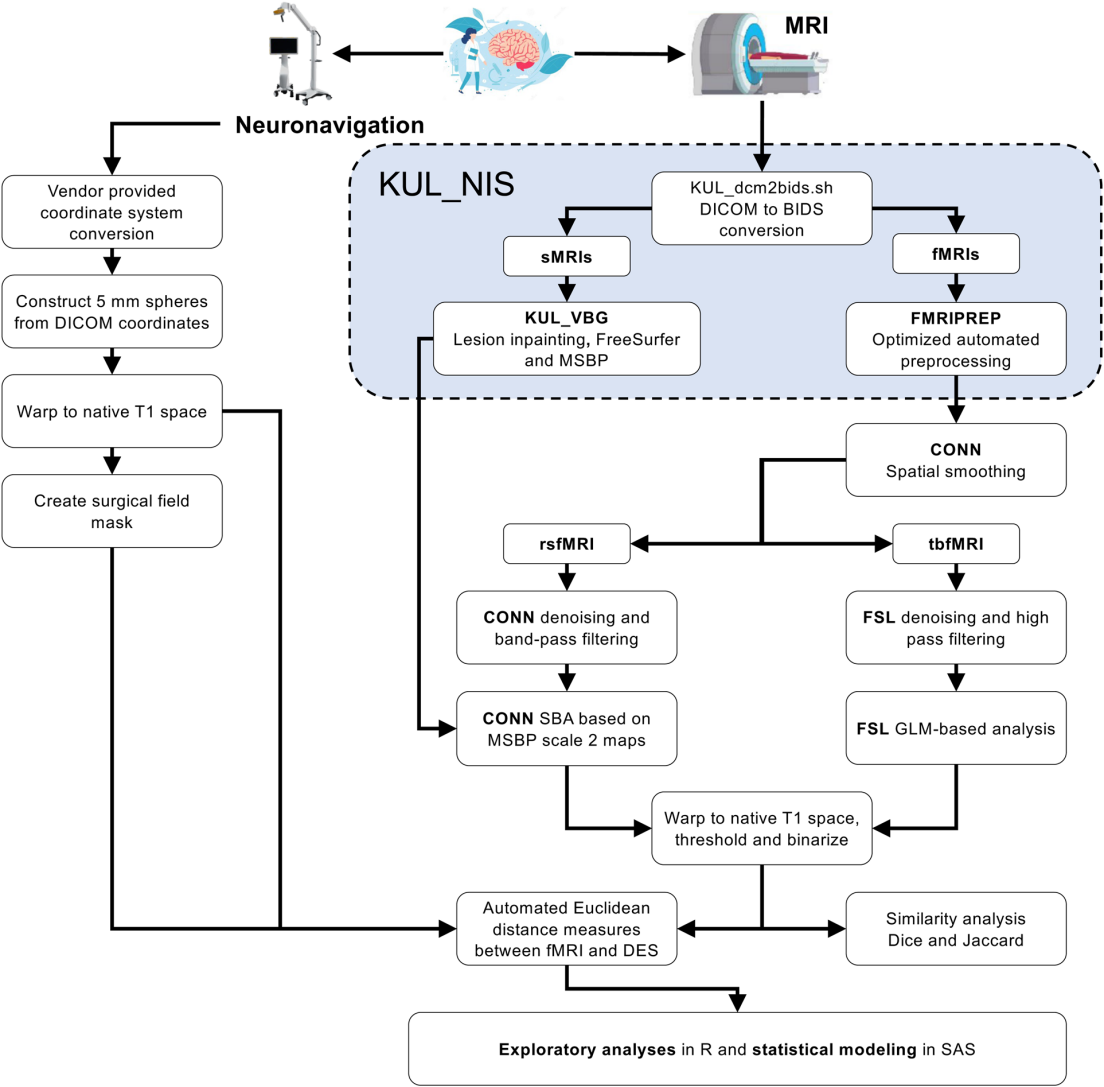
Schematic representation of the data preprocessing and analysis workflow used to compare different fMRI results with intraoperative mapping outcome. MRI = magnetic resonance imaging, KUL_NIS = KU Leuven neuroimaging suite, BIDS = brain imaging data structure, KUL_VBG = KU Leuven virtual brain grafting, CONN = functional connectivity toolbox, SBA = seed-based analysis, GLM = general linear model, DSC = dice similarity coefficient, JI = Jaccard index.

Figure 1shows a schematic representation of the data preprocessing and analysis workflow. Data conversion, lesion segmentation with ITK-snap v3.8.0 (Yushkevich et al., 2019), lesion inpainting with KU Leuven Virtual Brain Grafting v0.52 (KUL_VBG) (Radwan et al., 2021) (https://github.com/KUL-Radneuron/KUL_VBG), and parcellation were previously described (Radwan et al., 2024). Briefly, single class lesion masks including perilesional edema were created semiautomatically using image classification for larger lesions and manual delineation for smaller lesions. We constructed 5 mm radius spheres centered around each DES coordinate, then calculated minimum Euclidean distances between the center of gravity (COG) of each DES sphere and every voxel in the corresponding functional map. Additionally, we calculated similarity measures using dice similarity coefficient (DSC) and Jaccard index (JI) between corresponding functional maps from the four BOLD fMRI methods. All acquired images were converted to the brain imaging data structure (BIDS) (Gorgolewski et al., 2016) format using the*KULeuven Neuroimaging suite (KUL_NIS)*(2018/2022)(https://github.com/treanus/KUL_NIS) and*Dcm2bids*(2016/2022).

#### 3.6.1 BOLD fMRI data analysis

All BOLD fMRI data were preprocessed using fmriprep v20.2.6 (Esteban et al., 2019), which combines methods from different software packages in an optimized preprocessing pipeline. This corrected for slice-timing, motion artifacts, EPI-included image distortion, calculated various covariates for denoising, and applied intermodality warping between the BOLD and anatomical images, as well as normalization to the asymmetrical MNI152 nonlinear 2009 template with 2 mm isotropic voxels (MNI152NLin2009cAsym_res-2). The middle echo images (TE = 33 ms) of the mTE-rsfMRI series were also used separately as the sTE-rsfMRI 3 mm method, which perfectly matches the mTE-rsfMRI data in acquisition parameters, and patient state. Fmriprep was used for combining the different echoes of mTE-rsfMRI data into a single time-series (DuPre et al., 2021;Kundu et al., 2012,^2013^;Posse et al., 1999). All preprocessed BOLD fMRI data were imported in the functional connectivity analysis toolbox (CONN) (Whitfield-Gabrieli & Nieto-Castanon, 2012) and smoothed with a 3D gaussian kernel of 6 mm full-width at half-maximum. ANTs v2.3.0 (Avants et al., 2011;Tustison et al., 2021) was used to warp resulting maps to native T1 space. For quality assurance, the mean framewise displacement (FD) and temporal signal-to-noise ratios (tSNR) were evaluated for all fMRI scans.

#### 3.6.2 Task-based fMRI processing

Spatially smoothed tbfMRI images generated by CONN were brain extracted, then denoised by regressing out the normalized framewise displacement using fsl_glm FSL v6.0 (Jenkinson et al., 2012), and high-pass filtered using fslmaths with σ = 20 TRs. Results were analyzed using a general linear model (GLM) in fsl_glm with default settings other than specifying a double gamma HRF convolution. Output Z-score maps were then warped back and resampled to native T1 space for further analysis.

#### 3.6.3 Resting-state fMRI processing

Preprocessed rsfMRI (sTE and mTE) was denoised and analyzed using the seed-to-voxel approach in CONN (Whitfield-Gabrieli & Nieto-Castanon, 2012) and default covariates. Default band-pass filtering (0.001–0.01 Hz) was applied after nuisance regression. The MSBP (Tourbier et al., 2020) scale-2 parcellation maps were propagated into subject-specific binary gray matter masks using ANTs then imported to CONN to define the seeds for functional connectivity analysis. Seed-to-voxel maps for hands and feet were derived from second-level GLM analysis by calculating the average group-level connectivity maps. We used bilateral precentral part 3 labels as seeds for the hands, and bilateral paracentral labels for the feet. The resulting subject-specific beta maps from CONN’s second-level GLM were used for further analysis.

#### 3.6.4 Thresholding fMRI maps

Results of tbfMRI and rsfMRI processing were constrained by a smoothed subject-specific gray-matter tissue mask derived from the normalized T1-weighted images, then warped back to native T1 space using antsApplyTransforms. The resulting masked fMRI maps were then thresholded at a minimum of 0 to reject anticorrelations, which were not deemed relevant for this analysis, as we were only interested in voxels that showed increased activity in response to a task in tbfMRI or were positively functionally connected to the seeds used in rsfMRI. This also avoided skewing the resulting Kmeans maps toward lower values. ANTs ThresholdImage with Kmeans (K = 100) rescaled the positive intensities in all functional maps to similar values (1–101) for all subjects. This was followed by applying the following in-house empirically developed formula for automatic calculation of a minimum threshold for the Kmeans images, where T = minimum threshold value, m = mean value of nonzero voxels, and s = standard deviation of nonzero voxels. This thresholding strategy allowed automatic cleanup of all fMRI maps for all patients while maintaining sensitivity and specificity to functionally active/connected voxels. The resulting thresholded fMRI maps were binarized for further analysis.

#### 3.6.5 DES coordinates processing and distance measures

Saved DES coordinates were exported from the neuronavigator in the proprietary format Xbrain and converted to millimeters using a proprietary BrainLab script “Xbrain to points.” Spheres with 5 mm radii (Cochereau et al., 2016) were created around each DES coordinate with FSL (Jenkinson et al., 2012) in the same space as the anatomical image used during surgery, then warped to T1 space with ANTs (Avants et al., 2011;Tustison et al., 2021) for comparison with the fMRI results. Distance measures were initially limited to the surgical field using subject-specific binary voxel masks generated by summing all DES spheres, then binarizing the result and applying a 3D morphological dilation filter with σ = 15 mm using ANTs ImageMath, then masking the outcome with the binary brain mask. This served only to accelerate the calculation of minimum distances in patients who had fMRI results within the mask. The search was done in the full fMRI maps without masking if no positive fMRI voxels were found within the surgical field mask. Minimum Euclidean distances, defined as the direct shortest distance between two points in the same three-dimensional space, were calculated between all DES coordinates, represented by the COG of each DES sphere, and voxels of the corresponding fMRI maps in Python 3.8 using nibabel v3.2.2 (Brett et al., 2022), numpy v1.22.3 (C. R. Harris et al., 2020), and scipy v1.4.1 (Virtanen et al., 2020). Continuous distance measures were rounded to their closest integers (in millimeters) because increments smaller than a single anatomical image voxel (1 mm) were not considered meaningful.

#### 3.6.6 fMRI similarity measures

Dice similarity coefficient (DSC) and Jaccard index (JI) were calculated between functional maps for the hands and feet generated with tbfMRI and each rsfMRI method for the same subject, as well as between the rsfMRI methods using ANTS (Tustison et al., 2021) LabelOverlapMeasures. Differences in similarity measures between fMRI methods were explored using descriptive statistics.

#### 3.6.7 Statistical testing

##### 3.6.7.1 Exploratory analysis

Comparing different fMRI methods when a ground truth is present may be achieved with techniques typically employed to compare screening tests, such as confusion matrices. However, due to the small sample size, unequal number of DES samples, and functional maps per patient, we first plotted the distance measures (Patil, 2021) without imposing any cutoff for agreement**(RQ1)**. Then, to account for the unequal repetition of DES coordinates per subject, the distance measures were averaged for nDES and pDES separately, so that each patient had at most one pDES and one nDES measurement for hands and/or feet. These averaged measures were then used to create ROC (Robin et al., 2011) curves to explore differences in sensitivity, specificity, and to estimate distance cutoffs for further analysis of the unaveraged data**(RQ2)**. Different distance cutoffs were estimated based on the local maxima of the averaged tbfMRI data and used to evaluate the rsfMRI methods. DeLong tests were used for direct pairwise comparison of the ROC curves.

Next, we explored differences in binary agreement and disagreement at the ROC-determined distance cutoffs**(RQ3)**, excluding subjects with missing modalities (PT006 and PT010). Positive results were represented by fMRI-DES pairs with a distance less than the cutoff, true positives if involving pDES, and false positives if involving nDES coordinates. Negative results were represented by fMRI-DES pairs with a distance above the cutoff, true negatives if involving nDES and false negatives if involving pDES. Lastly, two-part linear models were used to compare all fMRI-DES distance measures between fMRI methods while accounting for the unequal intrasubject DES repetition**(RQ4)**at the different distance cutoffs determined on the tbfMRI ROC.

##### 3.6.7.2 Two-part linear modeling

Distance data thresholded at the three cutoffs determined by ROC of all pooled data were used for a two-part linear mixed model and posthoc testing to compare fMRI methods while accounting for the unequal number of DES coordinates between different subjects. The thresholded distances data were a semicontinuous variable with excess zeros and an extremely right-skewed distribution, violating assumptions of normality. Therefore, and given the within-subjects nesting of repeated distance measures, we opted for a two-part model for longitudinal data (Farewell et al., 2017;Tooze et al., 2002)**(RQ3)**. The model was estimated with the %MIXCORR macro provided byTooze et al. (2002)and PROC NLMIXED in SAS studio v9.4 (SAS Institute, Cary, NC, USA).

The first part (A) predicted the probability of overlap (distance = 0) and the second part (B) predicted the distances between nonoverlapping (distance > 0) fMRI-DES coordinate pairs. Distances were the dependent variable and DES response, and fMRI method (tbfMRI, sTE-rsfMRI 2 mm, sTE-rsfMRI 3 mm, and mTE-rsfMRI) was used as predictors in both parts of the model, and adaptive Hochberg’s (Hochberg & Benjamini, 1990) family-wise error rate (FWE) correction was used to control for type(I) error in posthoc testing. No covariates were used in this analysis due to the small sample size; further details can be found inSupplementary Material.

## 4 Results

### 4.1 Lesion segmentation

**Fig. 2.**
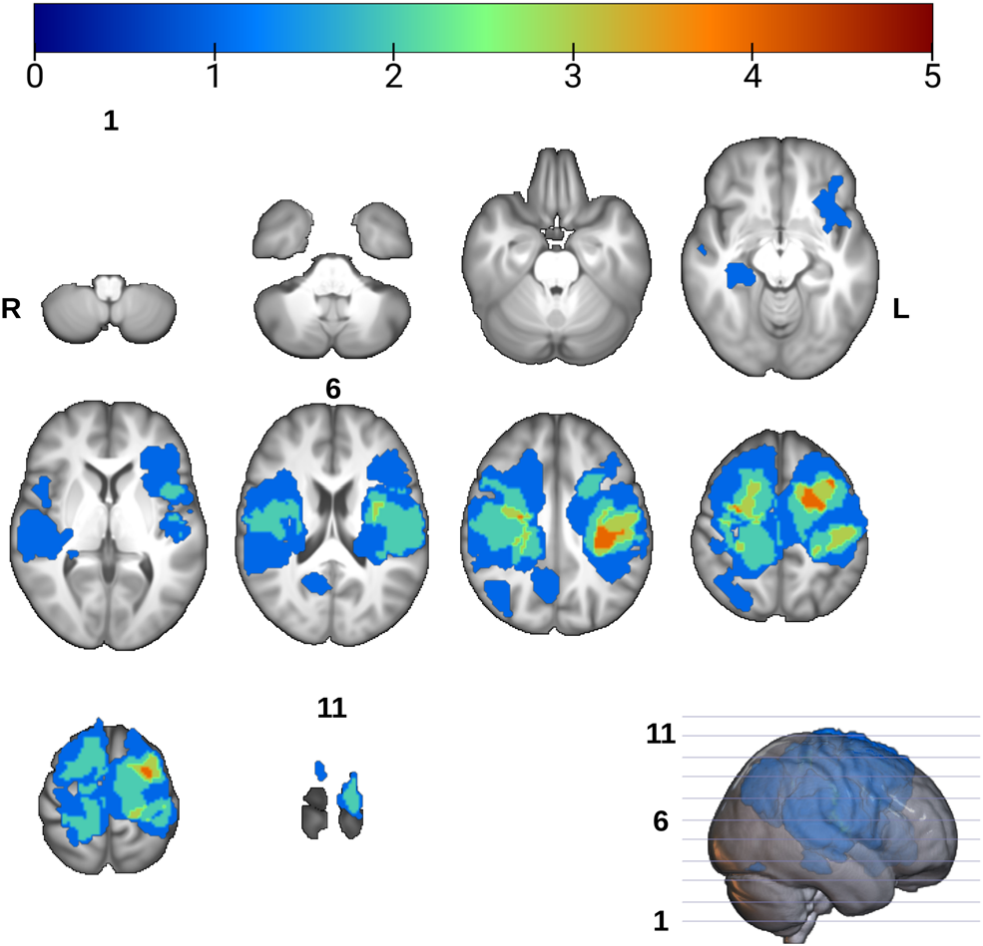
Spatial distribution of lesions from all patients overlaid onto the UK biobank T1 template brain in standard Montreal neurological institute (MNI) space. Overlay voxel intensities correspond to the sum of lesion masks occupying it. R = right, L = Left, slice numbers are indicated for the first, middle, and last slices.

Volumetric lesion voxel masks included perilesional edema in case of neoplasms. The median lesion volume was 44.70 ml, minimum = 1.20, maximum = 124.78, and IQR = 46.61 ml.Figure 2shows the cumulative voxel-wise lesion distribution maps in this sample of patients over the whole brain in the MNI152 space.

### 4.2 BOLD fMRI mapping

All fMRI data were successfully processed resulting in 22 tbfMRI maps, 22 sTE-rsfMRI maps, and 20 mTE-rsfMRIs as patients PT006 and PT010 did not undergo the mTE-rsfMRI scan. Hands were mapped for 16 patients using tbfMRI (15 with bilateral finger-tapping and unilateral fist-clenching for PT002), and bilaterally for all 16 patients from sTE-rsfMRI, and for 14 patients using mTE-rsfMRI, and feet were mapped bilaterally for 5 patients using the 4 methods, while PT006 and PT010 had no mTE-rsfMRI data. Task-based fMRI maps showed the expected pattern of activation in the primary sensory-motor cortex, premotor, and supplementary motor cortices, as well as occasional activity in the parietal proprioceptive cortex, and cerebellar activity.

Resting-state fMRI FC maps generated using bilateral primary sensory-motor cortical seeds showed a generally more widespread, and less specific pattern of connectivity, in most cases covering the areas of activity shown on tbfMRI maps, often extending beyond those areas and including FC within the opercular cortex and occipital lobes.

PT002, 005, 009, and 016 showed a visually worse outcome for sTE_3mm and mTE mapping for the hands. While for the feet the least specific outcome was found in PT005. PT002 performed unilateral left hand and left foot tasks, while PT005 performed a left foot task, as a result, the tbfMRI maps for these patients look the least similar to their resting-state counterparts. However, in both cases, most active areas in the unilateral task were also positive on rsfMRI though with notably less specificity.

**Fig. 3.**
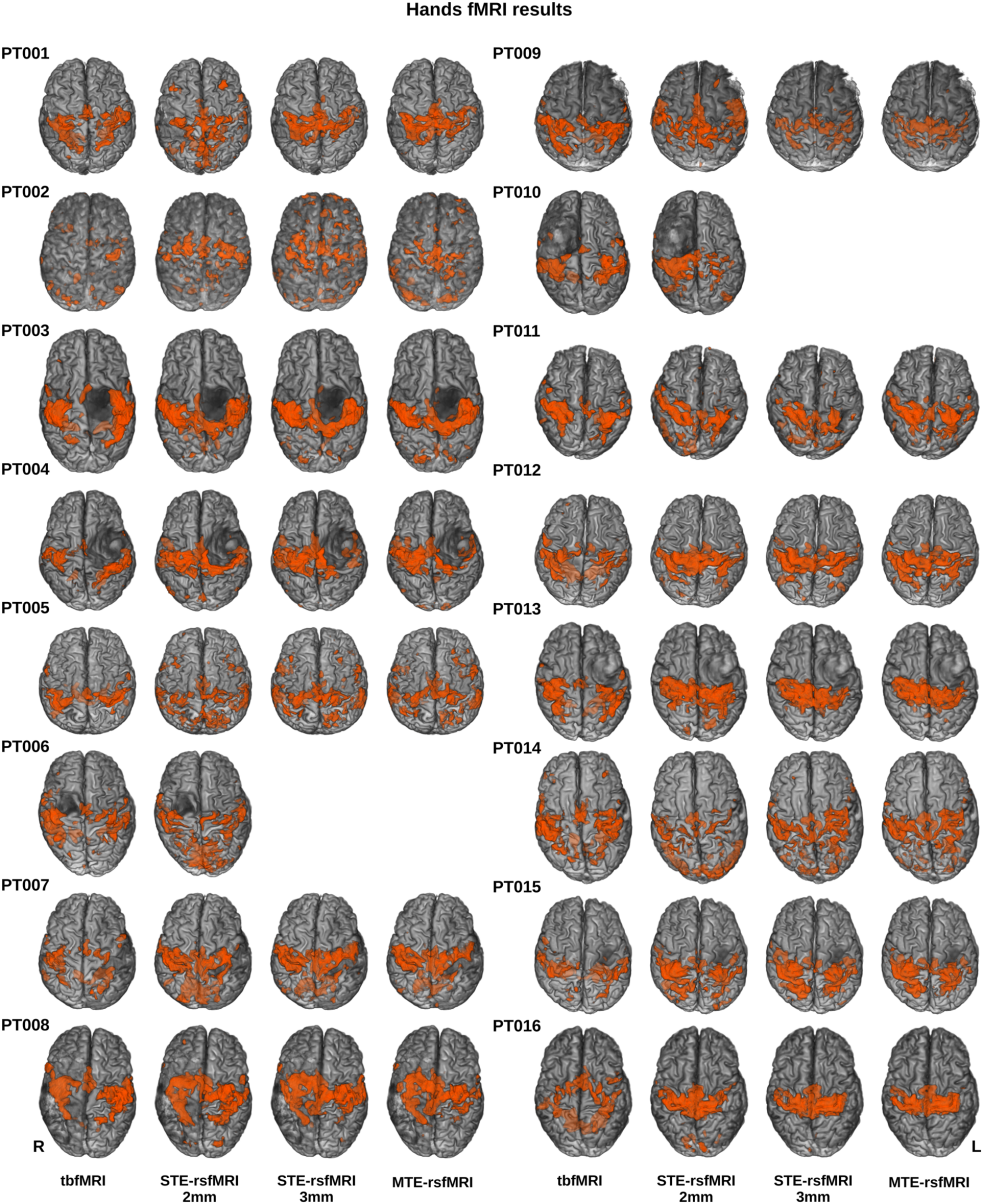
Thresholded results for hands fMRI mapping using all methods overlaid in orange on semitransparent surface-rendered T1 images for each patient in superior view, empty cells indicate a missing multiecho rsfMRI scan, tbfMRI = task-based fMRI, sTE-rsfMRI = single-echo resting-state fMRI, mTE-rsfMRI = multiecho rsfMRI.

Figures 3and4show all resulting fMRI maps for the hands and feet tasks, respectively. PT002 showed the highest mean FD of 3.6 mm on the tbfMRI and 2.7 mm on the mTE-rsfMRI data, however, did not notably differ from the rest of the patients in tSNR and upon visual inspection showed acceptable results despite increased false-positives. Thus, none of the fMRI maps were excluded from further analysis. Mean FD and tSNR values are plotted and shown inSupplementary Figures 1 and 2.

**Fig. 4.**
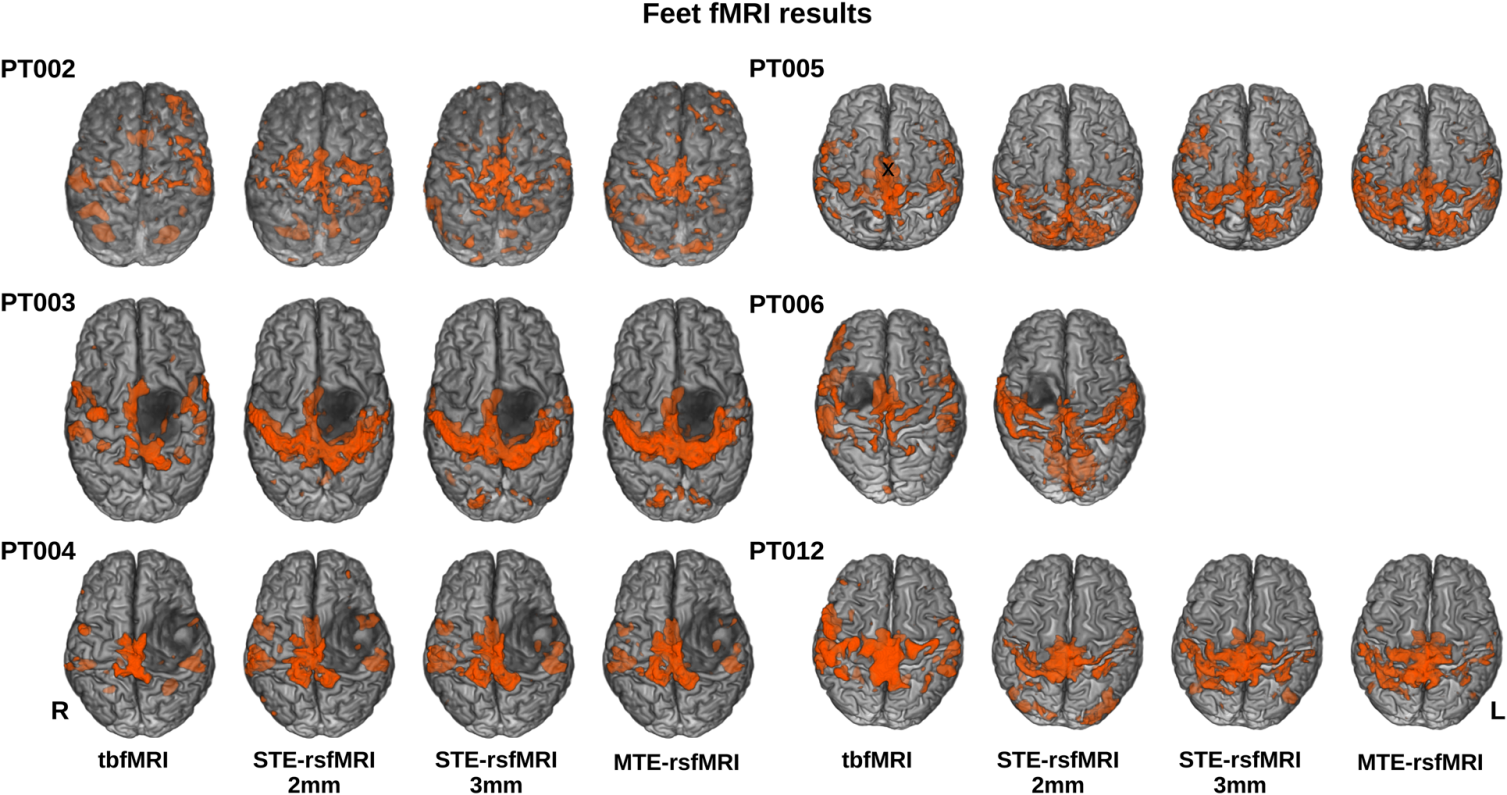
Thresholded results for feet fMRI mapping using all methods overlaid in orange on semitransparent surface-rendered T1 images for each patient in superior view, tbfMRI = task-based fMRI, sTE-rsfMRI = single-echo resting-state fMRI, mTE-rsfMRI = multiecho rsfMRI.

### 4.3 Intraoperative mapping and distance measures

**Fig. 5.**
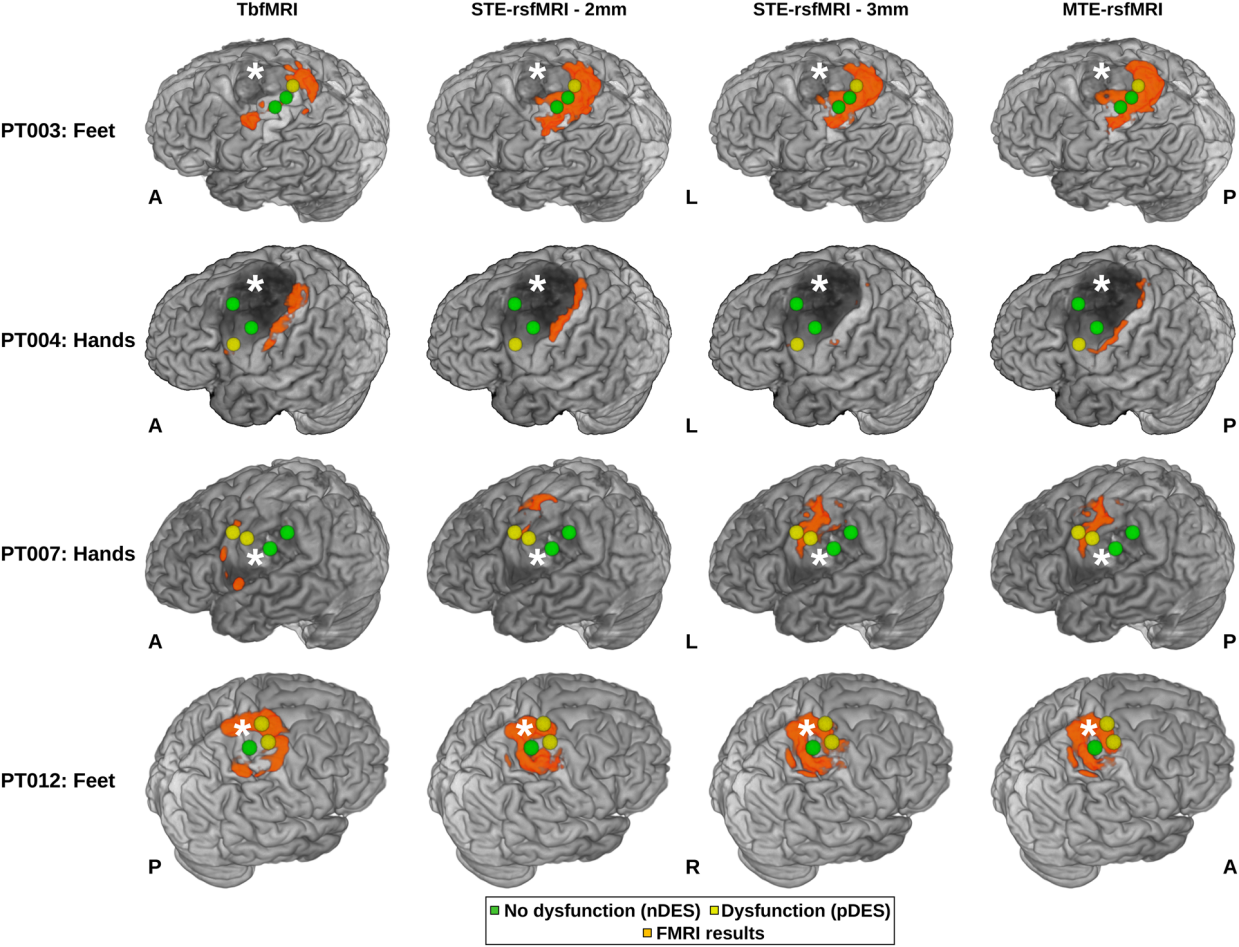
DES spheres and surgical field-masked fMRI mapping results for hands and feet with the three methods overlaid on top of semitransparent surface-rendered T1 images. Yellow spheres represent pDES, and green spheres indicate nDES, fMRI results are shown in orange. White asterisks indicate site of pathology, PT = patient, nDES = negative direct electrical stimulation, pDES = positive DES, tbfMRI = task-based fMRI, sTE-rsfMRI = single-echo resting-state fMRI, mTE-rsfMRI = multiecho rsfMRI, A = anterior, P = posterior, L = left, R = right, S = superior, I = inferior.

DES mapping resulted in a total of 23 positive DES (pDES) and 88 negative DES (nDES) coordinates.Figure 5shows images from exemplar patients demonstrating DES spheres and example functional mapping results included in the surgical field mask. Further details on results of intraoperative DES mapping can be found inSupplementary Table 2. Functional maps were paired with the relevant pDES spheres and all nDES spheres, which resulted in 512 distance measures in total with 138 measures for tbfMRI and sTE-rsfMRI at 2 mm and 118 for sTE-rsfMRI at 3 mm and mTE-rsfMRI. All included patients underwent awake motor mapping. Patients 001, 007, 008, and 009 had no pDES coordinates, that is, no responses were found at any of the saved coordinates on stimulation, while patients 003 and 012 had no nDES coordinates, that is, all saved DES coordinates were positive on stimulation. The saved pDES coordinates were tested against all positive fMRI voxels without distinguishing between the nature of the response, that is, primary sensory-motor, supplementary motor, or premotor areas, while the saved nDES coordinates were considered negative for both hands and feet. SeeSupplementary Figures 3 and 4for the DES spheres per patient and the consequent surgical field masks, as well asSupplementary Figure 5for plots of distance measures per DES response.

### 4.4 Similarity analysis

In terms of intermethod intrasubject similarity regardless of task, DSC and JI scores showed modest similarity between tbfMRIs and the three rsfMRI methods, with sTE-rsfMRI at 2 mm (DSC/JI median = 0.203/0.113, IQR = 0.159/0.103) scoring lower than mTE-rsfMRI (DSC/JI median = 0.260/0.150, IQR = 0.136/0.087) and sTE-rsfMRI at 3 mm (DSC/JI median = 0.284/0.166, IQR = 0.169/0.109). Higher similarity measures were found between sTE-rsfMRI at 2 mm and mTE (DSC/JI median = 0.536/0.367, IQR = 0.341/0.288), sTE-rsfMRI at 3 mm and mTE-rsfMRI (DSC/JI median = 0.557/0.728, IQR = 0.248/0.229), and sTE-rsfMRI at 2 mm and 3 mm (DSC/JI median = 0.546/0.376. IQR = 0.345/0.301). DSC and JI were found to be higher for the hands (DSC/JI median = 0.333/0.200, IQR = 0.358/0.326) than for feet (DSC/JI median = 0.269 /0.164, IQR = 0.306/0.348), seeSupplementary Table 3a and bfor more details.

### 4.5 Statistical testing

#### 4.5.1 Exploratory analysis

**Fig. 6.**
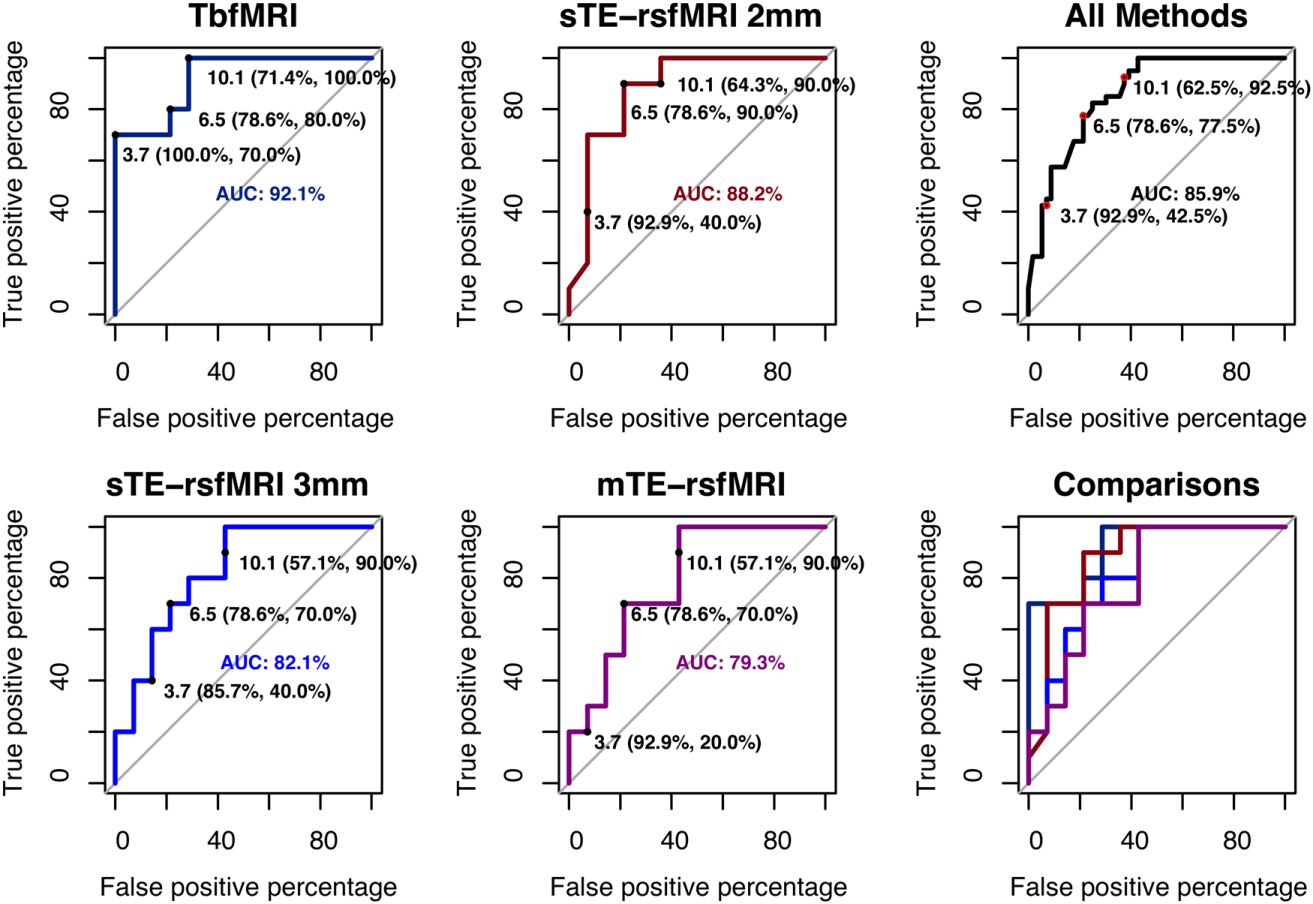
ROCs plots and distance cutoffs per fMRI method, the corresponding sensitivity and specificity values are shown as well as the area under the curve (AUC) value per method, tbfMRI = task-based fMRI, sTE-rsfMRI = single-echo resting-state fMRI, mTE-rsfMRI = multiecho rsfMRI.

Excluding PT006 and PT010, who did not undergo mTE-rsfMRI, only minor differences were found between fMRI methods for unthresholded distance measures**(RQ1)**, seeTable 4andSupplementary Figure 5. ROC curves local maxima determined three cutoffs of 3.7, 6.5, and 10.1 (integers: 4, 7, and 10) mm for the averaged tbfMRI data,**(RQ2)**seeTable 5andFigure 6for detailed results, andSupplementary Figure 6for ROCs generated from unaveraged distances. Briefly for the averaged data, tbfMRI had the highest sensitivity, specificity, and area under the curve, AUC = 92.1%, followed by sTE-rsfMRI at 2 mm, which had comparable sensitivity, mildly lower specificity, and AUC = 88.2%. The reduced voxel size seemed to induce a reduction in accuracy, as sTE-rsfMRI at 3 mm showed AUC = 82.1%, and mTE-rsfMRI scored the lowest, AUC = 79.3%. All methods showed lower sensitivity and higher specificity at the lowest cutoff, and higher sensitivity and lower specificity at higher cutoffs. DeLong tests showed no significant differences between any of the fMRI methods, seeSupplementary Table 4.

**Table 4.** Descriptive statistics for distance measures (in millimeters) per DES response, function, and fMRI method.

Function	DES response	fMRI method	Mean	StDev	Median	IQR	Var	Range	N
Hands	pDES	TbfMRI	4.176	3.225	4	6	10.404	10	17
		sTE-rsfMRI 2 mm	4.647	3.426	5	4	11.742	12	17
		sTE-rsfMRI 3 mm	4.933	4.0789	4	4	16.638	14	15
		mTE-rsfMRI	6.066	3.6344	6	4.5	13.209	13	15
	nDES	TbfMRI	14.534	10.085	12	14	101.722	45	88
		sTE-rsfMRI 2 mm	15.886	10.655	14	14.25	113.550	46	88
		sTE-rsfMRI 3 mm	15.389	10.917	13	16.75	119.183	38	76
		mTE-rsfMRI	15.618	10.527	13	17.25	110.825	42	76
Feet	pDES	TbfMRI	2.166	1.471	2.5	1.75	2.166	4	6
		sTE-rsfMRI 2 mm	2.666	2.160	2.5	2.5	4.666	6	6
		sTE-rsfMRI 3 mm	3.6	3.209	2	2	10.3	8	5
		mTE-rsfMRI	3.4	3.209	2	1	10.3	8	5
	nDES	TbfMRI	13.925	12.034	10	12	144.840	50	27
		sTE-rsfMRI 2 mm	13.851	12.024	9	16.5	144.592	37	27
		sTE-rsfMRI 3 mm	14.181	13.022	10.5	24	169.584	36	22
		mTE-rsfMRI	15.409	13.730	10	24.75	188.538	40	22

DES = direct electrical stimulation, pDES = positive DES, nDES = negative DES, fMRI = functional magnetic resonance imaging, StDev = standard deviation, IQR = interquartile range, Var = variance, N = number, tbfMRI = task-based fMRI, sTE-rsfMRI = single-echo resting-state fMRI, mTE-rsfMRI = multiecho resting-state fMRI.

**Table 5.** ROC-derived accuracy measures at different cutoffs.

ROC accuracy measures at different distance cutoffs 4/7/10 mm for averaged and raw distance measures				
	Sensitivity %		Specificity %	
Method	Averaged	Raw	Averaged	Raw
TbfMRI	70/80/100	56.5/78.3/100	100/78.6/71.4	88.7/73/56.5
STE-rsfMRI 2 mm	40/90/90	47.8/82.6/91.3	92.9/78.6/64.3	88.7/75.7/57.4
STE-rsfMRI 3 mm	40/90/90	45/80/90	85.7/78.6/57.1	84.7/70.4/54.1
MTE-rsfMRI	20/70/90	35/70/90	92.9/78.6/57.1	88.8/72.4/58.2

TbfMRI = task-based fMRI, STE-rsfMRI = single-echo resting-state fMRI, MTE-rsfMRI = multiecho resting-state fMRI.

**Fig. 7.**
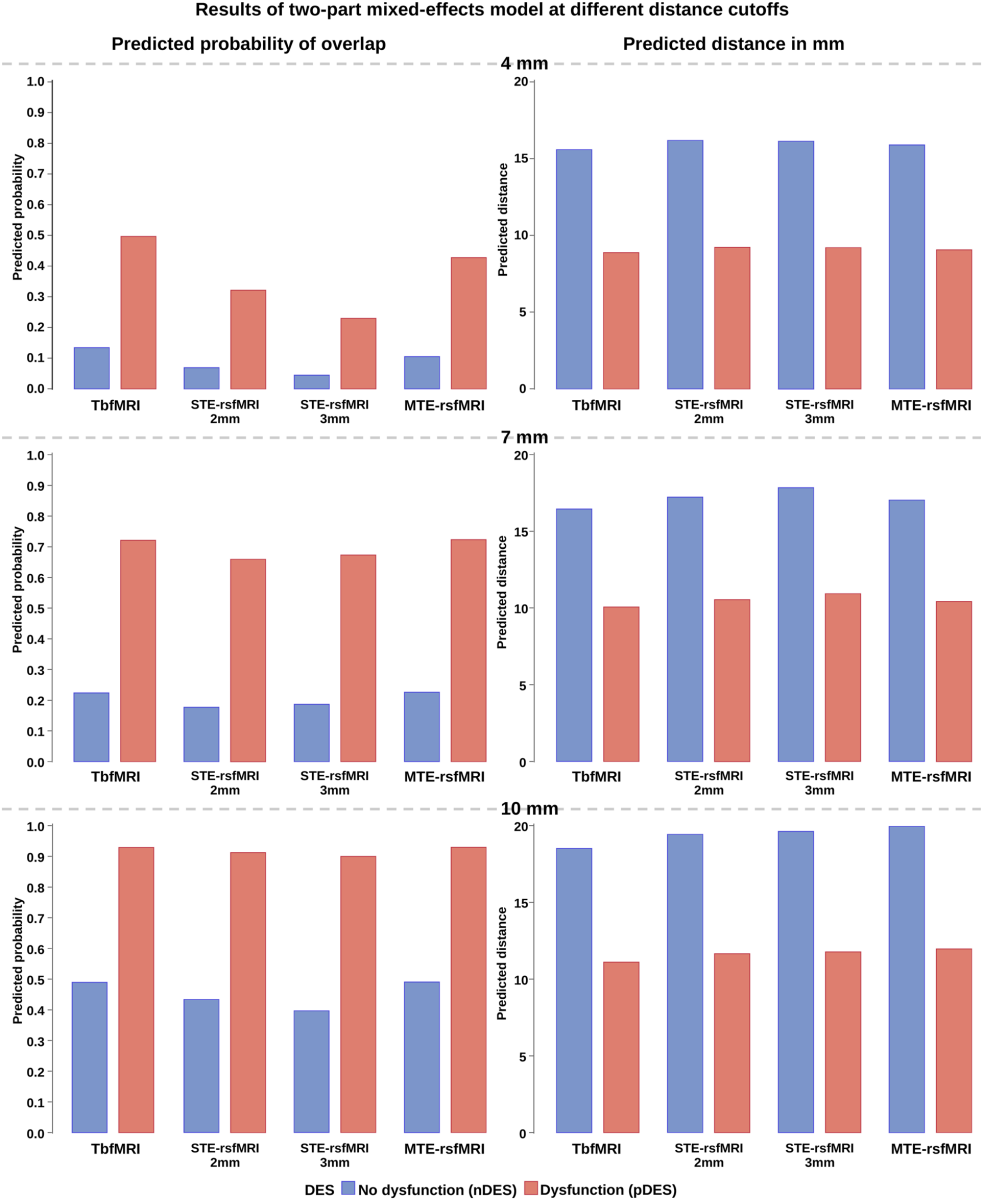
Results for predicted probability of overlap and predicted distances for nonoverlapping DES spheres averaged over fMRI tasks at different distance cutoffs for defining agreement, nDES = negative direct electrical stimulation, pDES = positive DES, tbfMRI = task-based fMRI, sTE-rsfMRI = single-echo resting-state fMRI, mTE-rsfMRI = multiecho rsfMRI.

Plots for binary agreement and disagreement rates showed only minor differences between fMRI methods at all ROC-determined distance cutoffs**(RQ3)**, seeSupplementary Figures 7 and 8.

#### 4.5.2 Two-part linear modeling

Below we describe the results of statistical modeling of predicted probability of agreement between fMRI-DES pairs where the distance measured was below the cutoff and predicted distance in case of disagreement (distance > cutoff)**(RQ4)**. Results of the logistic regression part (model A) are followed by the results of the lognormal part (model B) for the three ROC-determined distance cutoffs.

Differences between the four fMRI methods were not significant at any of the ROC-determined distance cutoffs, indicating that there were no significant differences in predicted probability of overlap and predicted distances between tbfMRI, sTE-rsfMRI (2 mm and 3 mm), and mTE-rsfMRI. Differences between DES response types were significant (p < 0.001) in both parts of the model at the three cutoffs, meaning that pDES coordinates were associated with significantly higher probabilities of overlap and shorter distances to fMRI activity regardless of the method used. No significant differences were found between hands and feet, indicating that the two domains behave similarly in both parts of the model. The results are listed inTable 6and illustrated visually inFigure 7.

**Table 6.** Summarized results of the estimated two-part models for predicting probability of overlap and distance between fMRI and DES.

Distance threshold for agreement			4 mm		7 mm		10 mm	
Parameter	Model	DF	t Value	Pr > |t|	t Value	Pr > |t|	t Value	Pr > |t|
tbfMRI v STE-rsfMRI 2 mm	A	14	-1.69	0.226	-0.78	0.980	-0.70	0.988
tbfMRI v STE-rsfMRI 3 mm	A	14	-0.64	0.531	0.03	0.980	-0.02	0.988
tbfMRI v mTE-rsfMRI	A	14	-2.49	0.052	-0.59	0.980	-1.02	0.988
STE-rsfMRI 2 mm v 3 mm	A	14	1.00	0.531	0.77	0.980	0.65	0.988
STE-rsfMRI 3 mm v MTE	A	14	-1.88	0.163	-0.60	0.98	-0.98	0.988
tbfMRI v STE-rsfMRI 2 mm	B	14	-0.52	0.963	-0.67	0.878	-0.72	0.881
tbfMRI v STE-rsfMRI 3 mm	B	14	-0.26	0.963	-0.47	0.878	-1.04	0.881
tbfMRI v mTE-rsfMRI	B	14	-0.45	0.963	-1.11	0.878	-0.84	0.881
STE-rsfMRI 2 mm v 3 mm	B	14	0.23	0.963	0.16	0.878	-0.38	0.881
STE-rsfMRI 3 mm v MTE	B	14	-0.18	0.963	-0.62	0.878	0.22	0.881

Model A = logistic regression part evaluating probability of overlap/agreement at distance cutoff, Model B = lognormal regression part evaluating distance measures in case of nonoverlap, DF = degrees of freedom, Pr > |t| = probability, TIV = total intracranial volume, sTE-rsfMRI = single-echo resting-state fMRI, mTE-rsfMRI = multiecho rsfMRI.

## 5 Discussion

The main aim of this study was to compare the accuracy of tbfMRI and rsfMRI using DES results as the ground truth. We did so first**(RQ1)**by comparing fMRI methods for the unthresholded distance measures. This showed minor differences between the fMRI methods for mapping hands and/or feet when compared with pDES and nDES. ROCs were then used to compare measures of accuracy, sensitivity, and specificity between the fMRI modalities**(RQ2)**. As tbfMRI represents the routine standard of practice for clinical fMRI mapping, its averaged distance values were used to estimate the distance cutoffs. This analysis showed that (a) tbfMRI was the most accurate while sTE-rsfMRI at 2 mm scored slightly worse. (b) The change in acquisition parameters, and patient state between the sTE-rsfMRI at 2 mm scan and the mTE-rsfMRI scan results in an apparent reduction in accuracy relative to DES. (c) The distance cutoff of 7 mm appeared to maximize both sensitivity and specificity on averaged data ROCs. Next, we compared fMRI methods for binary agreement measures at the three cutoffs**(RQ3)**, which also showed only minor differences between fMRI methods when compared with pDES and nDES. Similarly, the two-part mixed-effects linear model showed only minor and nonsignificant differences between the fMRI methods at the three distance cutoffs**(RQ4)**.

While mTE-rsfMRI could initially be expected to show the best performance among the rsfMRI acquisitions, this expectation is tempered when differences in acquisition parameters, and lack of randomization in scan order between tbfMRI, sTE, and mTE-rsfMRI are considered. Standardized preprocessing, resampling, and versatile statistical testing can only account for so much of the variance such differences could introduce. These differences, along with the rather low number of echoes acquired for mTE, could potentially account for its apparently lower performance on the ROC curves inFigure 6andSupplementary Figures 5 and 6, which was not reflected in the results of the two-part mixed-effects models at any of the distance cutoffs, as would be expected for a small difference. In retrospect, one of the most crucial differences between mTE and the other fMRI scans is the in-plane resolution, which could be expected to have a rather large effect, potentially meaning that mTE-rsfMRI could benefit from the use of lower thresholds than sTE-fMRI data.

The results of the similarity analysis with DSC and JI were in line with previous studies, which reported rather low agreement between tbfMRI and rsfMRI when comparing on whole brain level, despite good concordance with DES (Rosazza et al., 2014). Similarity scores can be expected to improve with matched acquisition parameters as, evidenced by the higher DSC and JI (0.557 and 0.728) between sTE-rsfMRI at 3 mm and mTE-rsfMRI than between sTE-rsfMRI at 2 mm and sTE-rsfMRI at 3 mm (0.536 and 0.367), or using a different seed selection for SBA, or with other functional analysis methods, for example, ICA (Branco et al., 2016). This may also be expected as tbfMRI and rsfMRI in fact measure different aspects of neural activity and, therefore, could be expected to give mildly similar but not identical results (Dierker et al., 2017). Considering the difference highlighted in the previous paragraph and the low similarity on the whole brain level, one could expect that it is much more likely to find a large difference in accuracy between the methods, a fact that lends more confidence to our results. Importantly, besides finding no significant differences in accuracy between tbfMRI and sTE-rsfMRI, these two behaved most similarly on the different plots and tests, suggesting that the sTE-rsfMRI is the most comparable with the tbfMRI for accurate SMN mapping.

While we also mapped language functions for some of the patients included in this work, due to the small sample size we focused only on mapping sensory-motor functions. However, the methods used here could potentially be applied to generate language resting-state maps resembling those of tbfMRI. Perhaps a promising approach could rely on the comparison of whole brain FC maps derived using the left-sided seeds, and another derived with the right-sided seeds, for example, in a laterality index fashion where instead of the hemispheric counts, the whole brain counts are used instead. (L_seed_whole_brain - R_seed_whole_brain)/(L_seed_whole_brain + R_seed_whole_brain). However, this was outside the scope of the current work.

This study adds to the growing body of evidence that presurgical functional brain mapping with rsfMRI is feasible with comparable accuracy with tbfMRI. In contrast to previous studies, here we used fully automated data analysis methods that accounted for the presence of pathology such as KUL_VBG (Radwan et al., 2021) for lesion inpainting and minimizing subsequent errors in resulting parcellation maps, as well as advanced statistical modeling. In addition, we generated hands- and feet-specific seed-based rsfMRI maps by relying on the finer-grained parcellation maps from MSBP (Tourbier et al., 2020), which, to the best of our knowledge, was previously done in only a few studies either using manual delineation (Rosazza et al., 2014;Schneider, 2015) or by repeating ICA within the SMN mask (Sohn et al., 2012). Only a small number of studies have included different fMRI methods and DES results as a gold standard in their analyses (Cui et al., 2022;Mitchell et al., 2013;Roland et al., 2019;Rosazza et al., 2014;Vakamudi et al., 2020;Zacà et al., 2018;Zhang et al., 2009), none of which used automated parcellation-based SBA for mapping hands and feet from rsfMRI, or included mTE-rsfMRI.

While the currently dominant paradigm in neurosurgical practice prioritizes mapping the eloquent sensory-motor, and/or language areas for preservation during surgery, recent evidence has shown the importance of mapping and preservation of functional networks that are generally thought of as noneloquent. Higher-order RSNs such as the ventral and dorsal attention networks, salience network, default mode network, and executive control networks if injured may be associated with reduced patient independence, and increased morbidity and mortality postoperatively (Dadario et al., 2021).

Multiple factors make rsfMRI a desirable supplement and/or potential substitute to tbfMRI in the clinical setting. First, the use of tbfMRI even for mapping functions requiring a relatively simple task such as sensory-motor and language tasks can be complicated, especially for clinical patients. RsfMRI, on the other hand, requires no task, and can provide functional maps for sensory-motor and language functions that resemble those of tbfMRI. In addition, rsfMRI can be used for mapping multiple functions from the same acquisition, including higher cognitive domains, such as attention, salience, and executive control, which typically require more challenging tasks to be mapped with tbfMRI. While some morphological differences remain between the maps produced by rsfMRI and tbfMRI in the literature as well in this study, this did not result in significant differences in distances calculated between DES and the different fMRI methods. Further research on this topic may help bridge this knowledge gap, particularly with further optimization of rsfMRI acquisitions and analysis methods.

Despite recent studies indicating that these RSNs can also be mapped from tbfMRI data by regressing out task-related signal changes (R. J. Harris et al., 2014;Pareto et al., 2018), there is an expectation of higher reliability of RSNs from rsfMRI compared with tbfMRI data analyzed with this approach. This expectation arises because a single rsfMRI scan typically acquires more time points (volumes) than a single tbfMRI scan, and a higher number of volumes have been correlated with increased reliability of mapped RSNs (Birn et al., 2013;White et al., 2014). It is important to note that while rsfMRI may be considered a viable alternative in case tbfMRI is not possible, if the patient is cooperative, tolerant to scanning, and if sufficient scan time is available, a combined acquisition of tbfMRI and rsfMRI remains a more data-rich approach. Additionally, analysis of task-independent signal change in tbfMRI data can still be expected to offer valuable information. In any case, the strengths and weaknesses of the functional mapping methods used should be clearly conveyed to the treating team of neurologists and neurosurgeons.

Lastly, the data analysis methods applied in this study may be further optimized and streamlined to allow for a shorter processing time, which would be more feasible in clinics. The integration of more advanced analytics into the clinical environment has been a matter of interest in the field for quite some time with minimal progress. Our preliminary tests of such research-oriented tools and their application in the clinic have shown a considerable gain in quality of results over commercially available clinically certified tools for task-based fMRI. Consequentially, this field of applicational development warrants further investment, in our opinion, in order to translate more sophisticated methodology to the clinical setting.

### 5.1 Study limitations

In contrast to recent studies (Dierker et al., 2017;Ngo et al., 2022;Niu et al., 2021;Parker Jones et al., 2017), we did not employ machine learning or deep learning methods for predicting tbfMRI from the rsfMRI data. While such studies show highly encouraging results, the majority are not easily accessible for clinical validation. Furthermore, these techniques typically require larger curated datasets for training and testing, and the pretrained models, if provided, might not translate easily to data from different scanners. Among the limitations of this study are the small sample size, nonstandardization of sensory-motor mapping tasks between fMRI and DES, patients sample heterogeneity in terms of age and pathology, as well as not including information on patient symptoms and postoperative follow-up. Lastly, mTE-fMRI was only evaluated as a resting-state technique, and only three TEs were acquired, while a higher number of TEs can be expected to improve mapping outcomes.

## 6 Conclusion

By using DES as the ground truth to compare measures of accuracy between tbfMRI, sTE-rsfMRI, and mTE-rsfMRI, we have demonstrated that automated parcellation-driven SBA sTE-rsfMRI can be used for presurgical brain mapping of sensory-motor representation of the hands and feet. Further investigation in a larger sample, preferably with denser sampling during invasive mapping, is necessary to explore the lower accuracy of mTE-rsfMRI and sTE-rsfMRI acquired at 3 mm, as well as the generalizability of these findings to different sites and different functional networks.

## Supplementary Material

Supplementary Material

## Data Availability

The data and code used for image processing and generating the results presented in this manuscript are not publicly available due to patient privacy, ethical limitations, and General Data Protection Regulations (GDPR) constraints, but can be obtained upon reasonable request from the corresponding author.

## Clinical Trials Registration

The manuscript is part of a cross‐sectional observational clinical study registered on clinicaltrials.gov with the identifier NCT06040580.

## References

[b1] Andersson , J. L. R. , Hutton , C. , Ashburner , J. , Turner , R. , & Friston , K. ( 2001 ). Modeling geometric deformations in EPI time series . NeuroImage , 13 ( 5 ), 903 – 919 . 10.1006/nimg.2001.0746 11304086

[b3] Avants , B. B. , Tustison , N. J. , Song , G. , Cook , P. A. , Klein , A. , Gee , J. C. , & Gee , C. ( 2011 ). A reproducible evaluation of ANTs similarity metric performance in brain image registration . NeuroImage , 54 ( 3 ), 2033 – 2044 . 10.1016/j.neuroimage.2010.09.025.A 20851191 PMC3065962

[b4] Bennett , C. M. , & Miller , M. B. ( 2013 ). fMRI reliability: Influences of task and experimental design . Cognitive, Affective, & Behavioral Neuroscience , 13 ( 4 ), 690 – 702 . 10.3758/s13415-013-0195-1 23934630

[b5] Birn , R. M. , Molloy , E. K. , Patriat , R. , Parker , T. , Meier , T. B. , Kirk , G. R. , Nair , V. A. , Meyerand , M. E. , & Prabhakaran , V. ( 2013 ). The effect of scan length on the reliability of resting-state fMRI connectivity estimates . NeuroImage , 83 , 550 – 558 . 10.1016/j.neuroimage.2013.05.099 23747458 PMC4104183

[b6] Biswal , B. , Yetkin , F. Z. , Haughton , V. M. , & Hyde , J. S. ( 1995 ). Functional connectivity in the motor cortex of resting human brain using echo-planar MRI . Magnetic Resonance in Medicine , 34 ( 4 ), 537 – 541 . 10.1002/mrm.1910340409 8524021

[b7] Branco , P. , Seixas , D. , Deprez , S. , Kovacs , S. , Peeters , R. , Castro , S. L. , & Sunaert , S. ( 2016 ). Resting-state functional magnetic resonance imaging for language preoperative planning . Frontiers in Human Neuroscience , 10 , 1 – 14 . 10.3389/fnhum.2016.00011 26869899 PMC4740781

[b8] Brett , M. , Markiewicz , C. J. , Hanke , M. , Côté , M.-A. , Cipollini , B. , McCarthy , P. , Jarecka , D. , Cheng , C. P. , Halchenko , Y. O. , Cottaar , M. , Larson , E. , Ghosh , S. , Wassermann , D. , Gerhard , S. , Lee , G. R. , Wang , H.-T. , Kastman , E. , Kaczmarzyk , J. , Guidotti , R. , … freec84 . ( 2022 ). nipy/nibabel: 3.2.2 [Computer software]. Zenodo. 10.5281/zenodo.6617121

[b9] Cochereau , J. , Deverdun , J. , Herbet , G. , Charroud , C. , Boyer , A. , Moritz-Gasser , S. , Bars Le , E., Molino , F. , Bonafé , A. , Menjot de Champfleur , N. , & Duffau , H. ( 2016 ). Comparison between resting state fMRI networks and responsive cortical stimulations in glioma patients . Human Brain Mapping , 37 ( 11 ), 3721 – 3732 . 10.1002/hbm.23270 27246771 PMC6867351

[b10] Cox , R. W. ( 1996 ). AFNI: Software for analysis and visualization of functional magnetic resonance neuroimages . Computers and Biomedical Research , 29 ( 3 ), 162 – 173 . 10.1006/cbmr.1996.0014 8812068

[b11] Cui , W. , Wang , Y. , Ren , J. , Hubbard , C. S. , Fu , X. , Fang , S. , Wang , D. , Zhang , H. , Li , Y. , Li , L. , Jiang , T. , & Liu , H. ( 2022 ). Personalized fMRI delineates functional regions preserved within brain tumors . Annals of Neurology , 91 ( 3 ), 353 – 366 . 10.1002/ana.26303 35023218 PMC9107064

[b12] Dadario , N. B. , Brahimaj , B. , Yeung , J. , & Sughrue , M. E. ( 2021 ). Reducing the cognitive footprint of brain tumor surgery . Frontiers in Neurology , 12 , 711646 . https://www.frontiersin.org/article/10.3389/fneur.2021.711646 34484105 10.3389/fneur.2021.711646 PMC8415405

[b13] Dcm2bids . ( 2022 ). [Python]. (Original work published 2016). UNF. https://github.com/UNFmontreal/Dcm2Bids

[b14] Dierker , D. , Roland , J. L. , Kamran , M. , Rutlin , J. , Hacker , C. D. , Marcus , D. S. , Milchenko , M. , Miller-Thomas , M. M. , Benzinger , T. L. , Snyder , A. Z. , Leuthardt , E. C. , & Shimony , J. S. ( 2017 ). Resting state fMRI in presurgical functional mapping: Sensorimotor localization . Neuroimaging Clinics of North America , 27 ( 4 ), 621 – 633 . 10.1016/j.nic.2017.06.011 28985933 PMC5773116

[b15] Duffau , H. , Capelle , L. , Sichez , J. , Faillot , T. , Abdennour , L. , Koune Law , D. J. , Dadoun , S. , Bitar , A. , Arthuis , F. , Van Effenterre , R. , & Fohanno , D. ( 1999 ). Intra-operative direct electrical stimulations of the central nervous system: The Salpêtrière experience with 60 patients . Acta Neurochirurgica , 141 ( 11 ), 1157 – 1167 . 10.1007/s007010050413 10592115

[b16] DuPre , E. , Salo , T. , Ahmed , Z. , Bandettini , P. A. , Bottenhorn , K. L. , Caballero-Gaudes , C. , Dowdle , L. T. , Gonzalez-Castillo , J. , Heunis , S. , Kundu , P. , Laird , A. R. , Markello , R. , Markiewicz , C. J. , Moia , S. , Staden , I. , Teves , J. B. , Uruñuela , E. , Vaziri-Pashkam , M. , Whitaker , K. , & Handwerker , D. A. ( 2021 ). TE-dependent analysis of multi-echo fMRI with *tedana* . Journal of Open Source Software , 6 ( 66 ), 3669 . 10.21105/joss.03669

[b17] Esteban , O. , Markiewicz , C. J. , Blair , R. W. , Moodie , C. A. , Isik , A. I. , Erramuzpe , A. , Kent , J. D. , Goncalves , M. , DuPre , E. , Snyder , M. , Oya , H. , Ghosh , S. S. , Wright , J. , Durnez , J. , Poldrack , R. A. , & Gorgolewski , K. J. ( 2019 ). fMRIPrep: A robust preprocessing pipeline for functional MRI . Nature Methods , 16 ( 1 ), Article 1. 10.1038/s41592-018-0235-4 PMC6319393 30532080

[b18] Fandino , J. , Kollias , S. S. , Wieser , H. G. , Valavanis , A. , & Yonekawa , Y. ( 1999 ). Intraoperative validation of functional magnetic resonance imaging and cortical reorganization patterns in patients with brain tumors involving the primary motor cortex . Journal of Neurosurgery , 91 ( 2 ), 238 – 250 . 10.3171/jns.1999.91.2.0238 10433312

[b19] Farewell , V. T. , Long , D. L. , Tom , B. D. M. , Yiu , S. , & Su , L. ( 2017 ). Two-part and related regression models for longitudinal data . Annual Review of Statistics and Its Application , 4 ( 1 ), 283 – 315 . 10.1146/annurev-statistics-060116-054131 PMC5590716 28890906

[b20] Fox , M. , & Greicius , M. ( 2010 ). Clinical applications of resting state functional connectivity . Frontiers in Systems Neuroscience , 4 , 19 . https://www.frontiersin.org/articles/10.3389/fnsys.2010.00019 20592951 10.3389/fnsys.2010.00019 PMC2893721

[b76] Friston , K. , Ashburner , J. , Kiebel , S. , Nichols , T. , & Penny , W. (Eds.). ( 2007 ). Statistical parametric mapping: The analysis of functional brain images . Academic Press . 10.1016/B978-0-12-372560-8.50052-8

[b21] Gonzalez-Castillo , J. , Kam , J. W. Y. , Hoy , C. W. , & Bandettini , P. A. ( 2021 ). How to interpret resting-state fMRI: Ask your participants . Journal of Neuroscience , 41 ( 6 ), 1130 – 1141 . 10.1523/JNEUROSCI.1786-20.2020 33568446 PMC7888219

[b22] Gorgolewski , K. J. , Auer , T. , Calhoun , V. D. , Craddock , R. C. , Das , S. , Duff , E. P. , Flandin , G. , Ghosh , S. S. , Glatard , T. , Halchenko , Y. O. , Handwerker , D. A. , Hanke , M. , Keator , D. , Li , X. , Michael , Z. , Maumet , C. , Nichols , B. N. , Nichols , T. E. , Pellman , J. , … Poldrack , R. A. ( 2016 ). The brain imaging data structure, a format for organizing and describing outputs of neuroimaging experiments . Scientific Data , 3 ( 1 ), 1 – 9 . 10.1038/sdata.2016.44 PMC4978148 27326542

[b23] Harris , C. R. , Millman , K. J. , van der Walt , S. J. , Gommers , R. , Virtanen , P. , Cournapeau , D. , Wieser , E. , Taylor , J. , Berg , S. , Smith , N. J. , Kern , R. , Picus , M. , Hoyer , S. , van Kerkwijk , M. H. , Brett , M. , Haldane , A. , del Río , J. F. , Wiebe , M. , Peterson , P. , … Oliphant , T. E. ( 2020 ). Array programming with NumPy . Nature , 585 , 357 – 362 . 10.1038/s41586-020-2649-2 32939066 PMC7759461

[b24] Harris , R. J. , Bookheimer , S. Y. , Cloughesy , T. F. , Kim , H. J. , Pope , W. B. , Lai , A. , Nghiemphu , P. L. , Liau , L. M. , & Ellingson , B. M. ( 2014 ). Altered functional connectivity of the default mode network in diffuse gliomas measured with pseudo-resting state fMRI . Journal of Neuro-Oncology , 116 ( 2 ), 373 – 379 . 10.1007/s11060-013-1304-2 24234804 PMC6763342

[b25] Hausman , H. K. , Hardcastle , C. , Kraft , J. N. , Evangelista , N. D. , Boutzoukas , E. M. , O’Shea , A. , Albizu , A. , Langer , K. , Van Etten , E. J. , Bharadwaj , P. K. , Song , H. , Smith , S. G. , Porges , E. , Hishaw , G. A. , Wu , S. , DeKosky , S. , Alexander , G. E. , Marsiske , M. , Cohen , R. , & Woods , A. J. ( 2022 ). The association between head motion during functional magnetic resonance imaging and executive functioning in older adults . NeuroImage: Reports , 2 ( 2 ), 100085 . 10.1016/j.ynirp.2022.100085 37377763 PMC10299743

[b26] Hochberg , Y. , & Benjamini , Y. ( 1990 ). More powerful procedures for multiple significance testing . Statistics in Medicine , 9 ( 7 ), 811 – 818 . 10.1002/sim.4780090710 2218183

[b27] Hsu , A.-L. , Hou , P. , Johnson , J. M. , Wu , C. W. , Noll , K. R. , Prabhu , S. S. , Ferguson , S. D. , Kumar , V. A. , Schomer , D. F. , Hazle , J. D. , Chen , J.-H. , & Liu , H.-L. ( 2018 ). IClinfMRI software for integrating functional MRI techniques in presurgical mapping and clinical studies . Frontiers in Neuroinformatics , 12 , 11 . 10.3389/fninf.2018.00011 29593520 PMC5854683

[b28] Hutton , C. , Bork , A. , Josephs , O. , Deichmann , R. , Ashburner , J. , & Turner , R. ( 2002 ). Image distortion correction in fMRI: A quantitative evaluation . NeuroImage , 16 ( 1 ), 217 – 240 . 10.1006/nimg.2001.1054 11969330

[b29] Jack , C. R. , Thompson , R. M. , Butts , R. K. , Sharbrough , F. W. , Kelly , P. J. , Hanson , D. P. , Riederer , S. J. , Ehman , R. L. , Hangiandreou , N. J. , & Cascino , G. D. ( 1994 ). Sensory motor cortex: Correlation of presurgical mapping with functional MR imaging and invasive cortical mapping . Radiology , 190 ( 1 ), 85 – 92 . 10.1148/radiology.190.1.8259434 8259434

[b30] Jenkinson , M. , Beckmann , C. F. , Behrens , T. E. J. , Woolrich , M. W. , & Smith , S. M. ( 2012 ). FSL . NeuroImage , 62 ( 2 ), 782 – 790 . 10.1016/j.neuroimage.2011.09.015 21979382

[b31] Kochiyama , T. , Morita , T. , Okada , T. , Yonekura , Y. , Matsumura , M. , & Sadato , N. ( 2005 ). Removing the effects of task-related motion using independent-component analysis . NeuroImage , 25 ( 3 ), 802 – 814 . 10.1016/j.neuroimage.2004.12.027 15808981

[b32] *KULeuven Neuroimaging Suite (KUL_NIS)* . ( 2022 ). [Shell, Python]. (Original work published 2018). https://github.com/treanus/KUL_NIS

[b33] Kundu , P. , Brenowitz , N. D. , Voon , V. , Worbe , Y. , Vértes , P. E. , Inati , S. J. , Saad , Z. S. , Bandettini , P. A. , & Bullmore , E. T. ( 2013 ). Integrated strategy for improving functional connectivity mapping using multiecho fMRI . Proceedings of the National Academy of Sciences of the United States of America , 110 ( 40 ), 16187 – 16192 . 10.1073/pnas.1301725110 24038744 PMC3791700

[b34] Kundu , P. , Inati , S. J. , Evans , J. W. , Luh , W.-M. , & Bandettini , P. A. ( 2012 ). Differentiating BOLD and non-BOLD signals in fMRI time series using multi-echo EPI . NeuroImage , 60 ( 3 ), 1759 – 1770 . 10.1016/j.neuroimage.2011.12.028 22209809 PMC3350785

[b35] Lee , M. H. , Miller-Thomas , M. M. , Benzinger , T. L. , Marcus , D. S. , Hacker , C. D. , Leuthardt , E. C. , & Shimony , J. S. ( 2016 ). Clinical resting-state fMRI in the preoperative setting: Are we ready for prime time ? Topics in Magnetic Resonance Imaging: TMRI , 25 ( 1 ), 11 – 18 . 10.1097/RMR.0000000000000075 26848556 PMC5640316

[b36] Lehéricy , S. , Duffau , H. , Cornu , P. , Capelle , L. , Pidoux , B. , Carpentier , A. , Auliac , S. , Clemenceau , S. , Sichez , J.-P. , Bitar , A. , Valery , C.-A. , Van Effenterre , R. , Faillot , T. , Srour , A. , Fohanno , D. , Philippon , J. , Bihan Le , D., & Marsault , C. ( 2000 ). Correspondence between functional magnetic resonance imaging somatotopy and individual brain anatomy of the central region: Comparison with intraoperative stimulation in patients with brain tumors . Journal of Neurosurgery , 92 ( 4 ), 589 – 598 . 10.3171/jns.2000.92.4.0589 10761647

[b37] Leuthardt , E. C. , Guzman , G. , Bandt , S. K. , Hacker , C. , Vellimana , A. K. , Limbrick , D. , Milchenko , M. , Lamontagne , P. , Speidel , B. , Roland , J. , Miller-Thomas , M. , Snyder , A. Z. , Marcus , D. , Shimony , J. , & Benzinger , T. L. S. ( 2018 ). Integration of resting state functional MRI into clinical practice—A large single institution experience . PLoS One , 13 ( 6 ), e0198349 . 10.1371/journal.pone.0198349 29933375 PMC6014724

[b38] Lu , J. , Zhang , H. , Hameed , N. U. F. , Zhang , J. , Yuan , S. , Qiu , T. , Shen , D. , & Wu , J. ( 2017 ). An automated method for identifying an independent component analysis-based language-related resting-state network in brain tumor subjects for surgical planning . Scientific Reports , 7 ( 1 ), 13769 – 13769 . 10.1038/s41598-017-14248-5 29062010 PMC5653800

[b39] Mitchell , T. J. , Hacker , C. D. , Breshears , J. D. , Szrama , N. P. , Sharma , M. , Bundy , D. T. , Pahwa , M. , Corbetta , M. , Snyder , A. Z. , Shimony , J. S. , & Leuthardt , E. C. ( 2013 ). A novel data-driven approach to preoperative mapping of functional cortex using resting-state functional magnetic resonance imaging . Neurosurgery , 73 ( 6 ), 969 – 983 . 10.1227/NEU.0000000000000141 24264234 PMC3871406

[b40] Morrison , M. A. , Churchill , N. W. , Cusimano , M. D. , Schweizer , T. A. , Das , S. , & Graham , S. J. ( 2016 ). Reliability of task-based fMRI for preoperative planning: A test-retest study in brain tumor patients and healthy controls . PLoS One , 11 ( 2 ), e0149547 . 10.1371/journal.pone.0149547 26894279 PMC4760755

[b41] Ngo , G. H. , Khosla , M. , Jamison , K. , Kuceyeski , A. , & Sabuncu , M. R. ( 2022 ). Predicting individual task contrasts from resting‐state functional connectivity using a surface‐based convolutional network . NeuroImage , 248 , 118849 . 10.1016/j.neuroimage.2021.118849 34965456 PMC10155599

[b42] Niskanen , E. , Könönen , M. , Villberg , V. , Nissi , M. , Ranta-aho , P. , Säisänen , L. , Karjalainen , P. , Äikiä , M. , Kälviäinen , R. , Mervaala , E. , & Vanninen , R. ( 2012 ). The effect of fMRI task combinations on determining the hemispheric dominance of language functions . Neuroradiology , 54 ( 4 ), 393 – 405 . 10.1007/s00234-011-0959-7 21932015 PMC3304062

[b43] Niu , C. , Wang , Y. , Cohen , A. D. , Liu , X. , Li , H. , Lin , P. , Chen , Z. , Min , Z. , Li , W. , Ling , X. , Wen , X. , Wang , M. , Thompson , H. P. , & Zhang , M. ( 2021 ). Machine learning may predict individual hand motor activation from resting-state fMRI in patients with brain tumors in perirolandic cortex . European Radiology , 31 ( 7 ), 5253 – 5262 . 10.1007/s00330-021-07825-w 33758954

[b44] O’Connor , E. E. , & Zeffiro , T. A. ( 2019 ). Why is clinical fMRI in a resting state? Frontiers in Neurology , 10 , 420 . 10.3389/fneur.2019.00420 31068901 PMC6491723

[b45] Pareto , D. , Sastre-Garriga , J. , Alonso , J. , Galán , I. , Arévalo , M. J. , Renom , M. , Montalban , X. , & Rovira , À. ( 2018 ). Classic block design “pseudo”-resting-state fMRI changes after a neurorehabilitation program in patients with multiple sclerosis . Journal of Neuroimaging: Official Journal of the American Society of Neuroimaging , 28 ( 3 ), 313 – 319 . 10.1111/jon.12500 29400912

[b46] Parker Jones , O. , Voets , N. L. , Adcock , J. E. , Stacey , R. , & Jbabdi , S. ( 2017 ). Resting connectivity predicts task activation in pre-surgical populations . NeuroImage: Clinical , 13 , 378 – 385 . 10.1016/j.nicl.2016.12.028 28123949 PMC5222953

[b47] Patil , I. ( 2021 ). Visualizations with statistical details: The “ggstatsplot” approach . Journal of Open Source Software , 6 ( 61 ), 3167 . 10.21105/joss.03167

[b48] Posse , S. , Wiese , S. , Gembris , D. , Mathiak , K. , Kessler , C. , Grosse-Ruyken , M.-L. , Elghahwagi , B. , Richards , T. , Dager , S. R. , & Kiselev , V. G. ( 1999 ). Enhancement of BOLD-contrast sensitivity by single-shot multi-echo functional MR imaging . Magnetic Resonance in Medicine , 42 ( 1 ), 87 – 97 . 10.1002/(SICI)1522-2594(199907)42:1<87::AID-MRM13>3.0.CO;2-O 10398954

[b49] Pur , D. R. , Eagleson , R. , Lo , M. , Jurkiewicz , M. T. , Andrade , A. , & de Ribaupierre , S. ( 2021 ). Presurgical brain mapping of the language network in pediatric patients with epilepsy using resting-state fMRI . Journal of Neurosurgery: Pediatrics , 27 ( 3 ), 259 – 268 . 10.3171/2020.8.PEDS20517 33418528

[b50] Qiu , T.-m. , Yan , C.-g. , Tang , W.-j. , Wu , J.-s. , Zhuang , D.-x. , Yao , C.-j. , Lu , J.-f. , Zhu , F.-p. , Mao , Y. , & Zhou , L.-f. ( 2014 ). Localizing hand motor area using resting-state fMRI: Validated with direct cortical stimulation . Acta Neurochirurgica , 156 ( 12 ), 2295 – 2302 . 10.1007/s00701-014-2236-0 25246146

[b51] Radwan , A. M. , Emsell , L. , Blommaert , J. , Zhylka , A. , Kovacs , S. , Theys , T. , Sollmann , N. , Dupont , P. , & Sunaert , S. ( 2021 ). Virtual brain grafting: Enabling whole brain parcellation in the presence of large lesions . NeuroImage , 229 , 117731 . 10.1016/j.neuroimage.2021.117731 33454411

[b52] Radwan , A. M. , Emsell , L. , Vansteelandt , K. , Cleeren , E. , Peeters , R. , De Vleeschouwer , S. , Theys , T. , Dupont , P. , & Sunaert , S. ( 2024 ). Comparative validation of automated presurgical tractography based on constrained spherical deconvolution and diffusion tensor imaging with direct electrical stimulation . Human Brain Mapping , 45 ( 6 ), e26662 . 10.1002/hbm.26662 38646998 PMC11033921

[b53] Robin , X. , Turck , N. , Hainard , A. , Tiberti , N. , Lisacek , F. , Sanchez , J.-C. , & Müller , M. ( 2011 ). pROC: An open-source package for R and S+ to analyze and compare ROC curves . BMC Bioinformatics , 12 ( 1 ), 77 . 10.1186/1471-2105-12-77 21414208 PMC3068975

[b54] Roland , J. L. , Hacker , C. D. , Snyder , A. Z. , Shimony , J. S. , Zempel , J. M. , Limbrick , D. D. , Smyth , M. D. , & Leuthardt , E. C. ( 2019 ). A comparison of resting state functional magnetic resonance imaging to invasive electrocortical stimulation for sensorimotor mapping in pediatric patients . NeuroImage: Clinical , 23 , 101850 . 10.1016/j.nicl.2019.101850 31077983 PMC6514367

[b55] Rosazza , C. , Aquino , D. , D’Incerti , L. , Cordella , R. , Andronache , A. , Zacà , D. , Bruzzone , M. G. , Tringali , G. , & Minati , L. ( 2014 ). Preoperative mapping of the sensorimotor cortex: Comparative assessment of task-based and resting-state fMRI . PLoS One , 9 ( 6 ), e98860 . 10.1371/journal.pone.0098860 24914775 PMC4051640

[b56] Schneider , F. C. , Pailler , M. , Faillenot , I. , Vassal , F. , Guyotat , J. , Barral , F. G. , & Boutet , C. ( 2015 ). Presurgical assessment of the sensorimotor cortex using resting-state fMRI . American Journal of Neuroradiology , 37 ( 1 ), 101 – 107 . 10.3174/ajnr.a4472 26381564 PMC7960206

[b57] Sohn , W. , Yoo , K. s , & Jeong , Y. ( 2012 ). Independent component analysis of localized resting-state functional magnetic resonance imaging reveals specific motor subnetworks . Brain Connectivity , 2 , 218 – 224 . 10.1089/brain.2012.0079 22738280

[b58] Stippich , C. , Rapps , N. , Dreyhaupt , J. , Durst , A. , Kress , B. , Nennig , E. , Tronnier , V. M. , & Sartor , K. ( 2007 ). Localizing and lateralizing language in patients with brain tumors: Feasibility of routine preoperative functional MR imaging in 81 consecutive patients 1 . Radiology , 243 ( 3 ), 828 – 836 . 10.1148/radiol.2433060068 17517936

[b59] Sunaert , S. ( 2006 ). Presurgical planning for tumor resectioning . Journal of Magnetic Resonance Imaging , 23 ( 6 ), 887 – 905 . 10.1002/jmri.20582 16649210

[b60] Tooze , J. A. , Grunwald , G. K. , & Jones , R. H. ( 2002 ). Analysis of repeated measures data with clumping at zero . Statistical Methods in Medical Research , 11 ( 4 ), 341 – 355 . 10.1191/0962280202sm291ra 12197301

[b61] Tourbier , S. , Alemán-Gómez , Y. , Griffa , A. , Cuadra , M. B. , & Hagmann , P. ( 2020 ). Multi-scale brain parcellator: A BIDS app for the lausanne connectome parcellation . F1000Research , 9 , 1714 . 10.7490/f1000research.1117844.1

[b62] Tustison , N. J. , Cook , P. A. , Holbrook , A. J. , Johnson , H. J. , Muschelli , J. , Devenyi , G. A. , Duda , J. T. , Das , S. R. , Cullen , N. C. , Gillen , D. L. , Yassa , M. A. , Stone , J. R. , Gee , J. C. , & Avants , B. B. ( 2021 ). The ANTsX ecosystem for quantitative biological and medical imaging . Scientific Reports , 11 ( 1 ), 9068 . 10.1038/s41598-021-87564-6 33907199 PMC8079440

[b63] Unadkat , P. , Fumagalli , L. , Rigolo , L. , Vangel , M. G. , Young , G. S. , Huang , R. , Mukundan , S. , Golby , A. , & Tie , Y. ( 2019 ). Functional MRI task comparison for language mapping in neurosurgical patients . Journal of Neuroimaging , 29 ( 3 ), 348 – 356 . 10.1111/jon.12597 30648771 PMC6506353

[b64] Vakamudi , K. , Posse , S. , Jung , R. , Cushnyr , B. , & Chohan , M. O. ( 2020 ). Real‐time presurgical resting‐state fMRI in patients with brain tumors: Quality control and comparison with task‐fMRI and intraoperative mapping . Human Brain Mapping , 41 ( 3 ), 797 – 814 . 10.1002/hbm.24840 31692177 PMC7268088

[b65] Van , A. N. , Montez , D. F. , Laumann , T. O. , Suljic , V. , Madison , T. , Baden , N. J. , Ramirez-Perez , N. , Scheidter , K. M. , Monk , J. S. , Whiting , F. I. , Adeyemo , B. , Chauvin , R. J. , Krimmel , S. R. , Metoki , A. , Rajesh , A. , Roland , J. L. , Salo , T. , Wang , A. , Weldon , K. B. , … Dosenbach , N. U. F. ( 2023 ). Framewise multi-echo distortion correction for superior functional MRI . bioRxiv , 2023.11.28.568744. 10.1101/2023.11.28.568744 PMC13224312 42232073

[b66] Virtanen , P. , Gommers , R. , Oliphant , T. E. , Haberland , M. , Reddy , T. , Cournapeau , D. , Burovski , E. , Peterson , P. , Weckesser , W. , Bright , J. , van der Walt , S. J. , Brett , M. , Wilson , J. , Millman , K. J. , Mayorov , N. , Nelson , A. R. J. , Jones , E. , Kern , R. , Larson , E. , … van Mulbregt , P. ( 2020 ). SciPy 1.0: Fundamental algorithms for scientific computing in Python . Nature Methods , 17 ( 3 ), 261 – 272 . 10.1038/s41592-019-0686-2 32015543 PMC7056644

[b67] Vysotski , S. , Madura , C. , Swan , B. , Holdsworth , R. , Lin , Y. , Munoz Del Rio , A. , Wood , J. , Kundu , B. , Penwarden , A. , Voss , J. , Gallagher , T. , Nair , V. A. , Field , A. , Garcia-Ramos , C. , Meyerand , M. E. , Baskaya , M. , Prabhakaran , V. , & Kuo , J. S. ( 2018 ). Preoperative FMRI associated with decreased mortality and morbidity in brain tumor patients . Interdisciplinary Neurosurgery , 13 , 40 – 45 . 10.1016/j.inat.2018.02.001 31341789 PMC6653633

[b68] Wengenroth , M. , Blatow , M. , Guenther , J. , Akbar , M. , Tronnier , V. M. , & Stippich , C. ( 2011 ). Diagnostic benefits of presurgical fMRI in patients with brain tumours in the primary sensorimotor cortex . European Radiology , 21 ( 7 ), 1517 – 1525 . 10.1007/s00330-011-2067-9 21271252 PMC3101350

[b69] White , T. , Muetzel , R. , Schmidt , M. , Langeslag , S. J. E. , Jaddoe , V. , Hofman , A. , Calhoun , V. D. , Verhulst , F. C. , & Tiemeier , H. ( 2014 ). Time of acquisition and network stability in pediatric resting-state functional magnetic resonance imaging . Brain Connectivity , 4 ( 6 ), 417 – 427 . 10.1089/brain.2013.0195 24874884 PMC4120810

[b70] Whitfield-Gabrieli , S. , & Nieto-Castanon , A. ( 2012 ). Conn: A functional connectivity toolbox for correlated and anticorrelated brain networks . Brain Connectivity , 2 ( 3 ), 125 – 141 . 10.1089/brain.2012.0073 22642651

[b71] Xie , J. , Chen , X. , Jiang , T. , Li , S. , Li , Z. , Zhang , Z. , Dai , J. , & Wang , Z. ( 2008 ). Preoperative blood oxygen level-dependent functional magnetic resonance imaging in patients with gliomas involving the motor cortical areas . Chinese Medical Journal , 121 ( 7 ), 631 – 635 . 10.1097/00029330-200804010-00011 18466684

[b72] Yushkevich , P. A. , Pashchinskiy , A. , Oguz , I. , Mohan , S. , Schmitt , J. E. , Stein , J. M. , Zukić , D. , Vicory , J. , McCormick , M. , Yushkevich , N. , Schwartz , N. , Gao , Y. , & Gerig , G. ( 2019 ). User-guided segmentation of multi-modality medical imaging datasets with ITK-SNAP . Neuroinformatics , 17 ( 1 ), 83 – 102 . 10.1007/s12021-018-9385-x 29946897 PMC6310114

[b73] Zacà , D. , Jovicich , J. , Corsini , F. , Rozzanigo , U. , Chioffi , F. , & Sarubbo , S. ( 2018 ). ReStNeuMap: A tool for automatic extraction of resting-state functional MRI networks in neurosurgical practice . Journal of Neurosurgery , 131 ( 3 ), 764 – 771 . 10.3171/2018.4.JNS18474 30485221

[b74] Zangaladze , A. , Sharan , A. , Evans , J. , Wyeth , D. H. , Wyeth , E. G. , Tracy , J. I. , Chervoneva , I. , & Sperling , M. R. ( 2008 ). The effectiveness of low-frequency stimulation for mapping cortical function . Epilepsia , 49 ( 3 ), 481 – 487 . 10.1111/j.1528-1167.2007.01307.x 17868054

[b75] Zhang , D. , Johnston , J. M. , Fox , M. D. , Leuthardt , E. C. , Grubb , R. L. , Chicoine , M. R. , Smyth , M. D. , Snyder , A. Z. , Raichle , M. E. , & Shimony , J. S. ( 2009 ). Preoperative sensorimotor mapping in brain tumor patients using spontaneous fluctuations in neuronal activity imaged with fMRI: Initial experience . Neurosurgery , 65 ( 6 Suppl. ), 226 – 236 . 10.1227/01.NEU.0000350868.95634.CA 19934999 PMC2796594

