# Supplementary material for: A comparison of fMRI presurgical mapping techniques with intraoperative brain mapping-based validation
Source: Imaging Neurosci (Camb). 2024 Aug 29;2:imag-2-00280. doi: 10.1162/imag_a_00280 (PMC12290823; doi:10.1162/imag_a_00280)
Supplement: Supplementary Material [file imag_a_00280-supp.pdf]

A comparison of fMRI presurgical mapping techniques with intraoperative brain mapping-based validation

Ahmed M. Radwan [1,2], Louise Emsell [1,2,3,4], Kristof Vansteelandt [2,3,4], Evy Cleeren [5], Ronald Peeters [6], Steven De Vleeschouwer [2,7,8], Tom Theys [2,7,8], Patrick Dupont [2,9], Stefan Sunaert [1,2,6]

Supplementary information

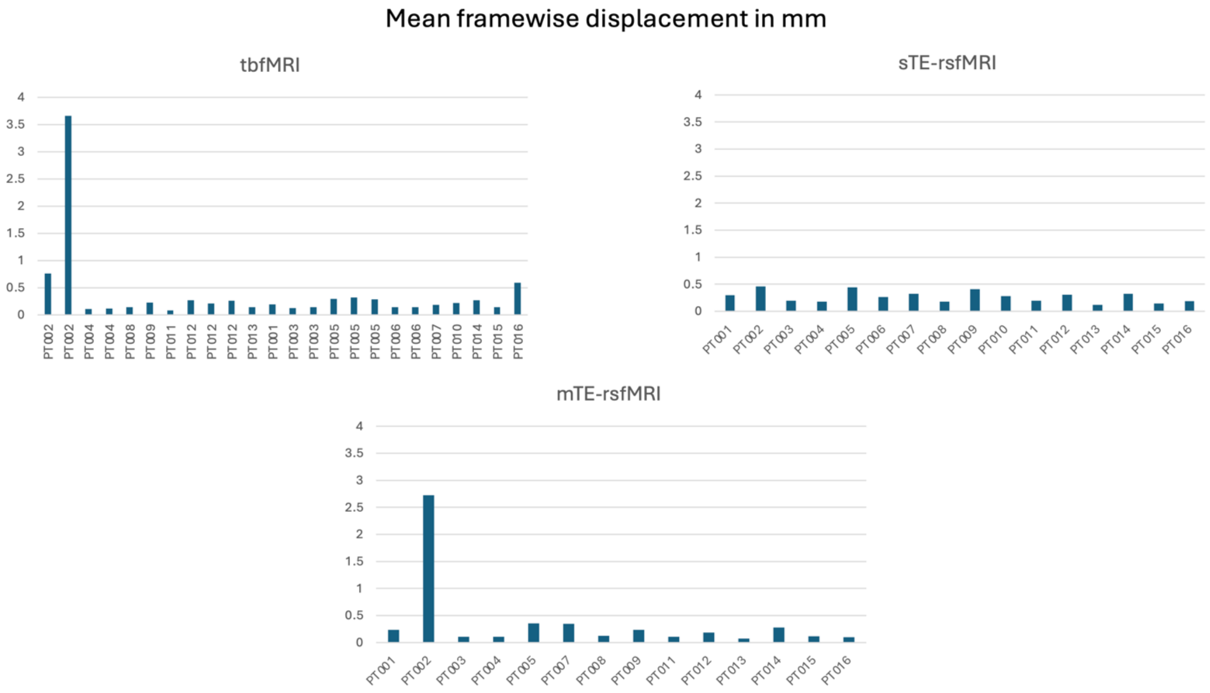

Supplementary figure 1: Column plots showing the mean framewise displacement per patient per fMRI scan type. tbfMRI = task-based functional MRI, sTE-rsfMRI = single-echo resting-state fMRI, mTE-rsfMRI = multi-echo rsfMRI

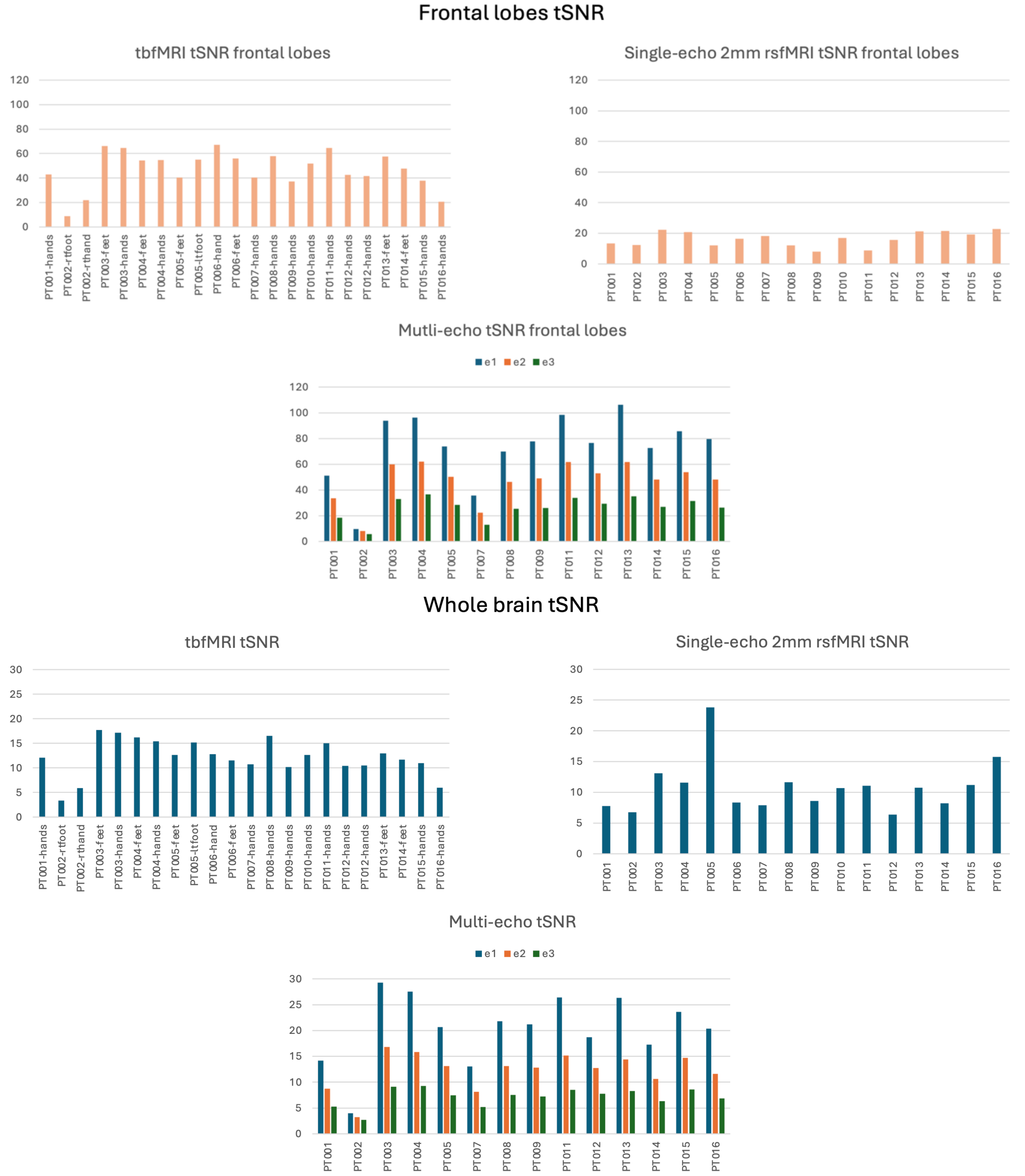

Supplementary figure 2: Column plots showing the mean temporal signal-to-noise ratio (tSNR) values for each patient and fMRI scanning method. The top panel shows tSNR for the frontal lobes only and the bottom panel shows the tSNR for the whole brain images. For the mTE-rsfMRI data, each echo's tSNR score is shown using a different color, blue is the first echo (TE = 8 ms), orange is the second echo (TE = 33 ms) and green is the last echo (TE = 58 ms). tbfMRI = task-based functional MRI, sTE-rsfMRI = single-echo resting-state fMRI, mTE-rsfMRI = multi-echo rsfMRI

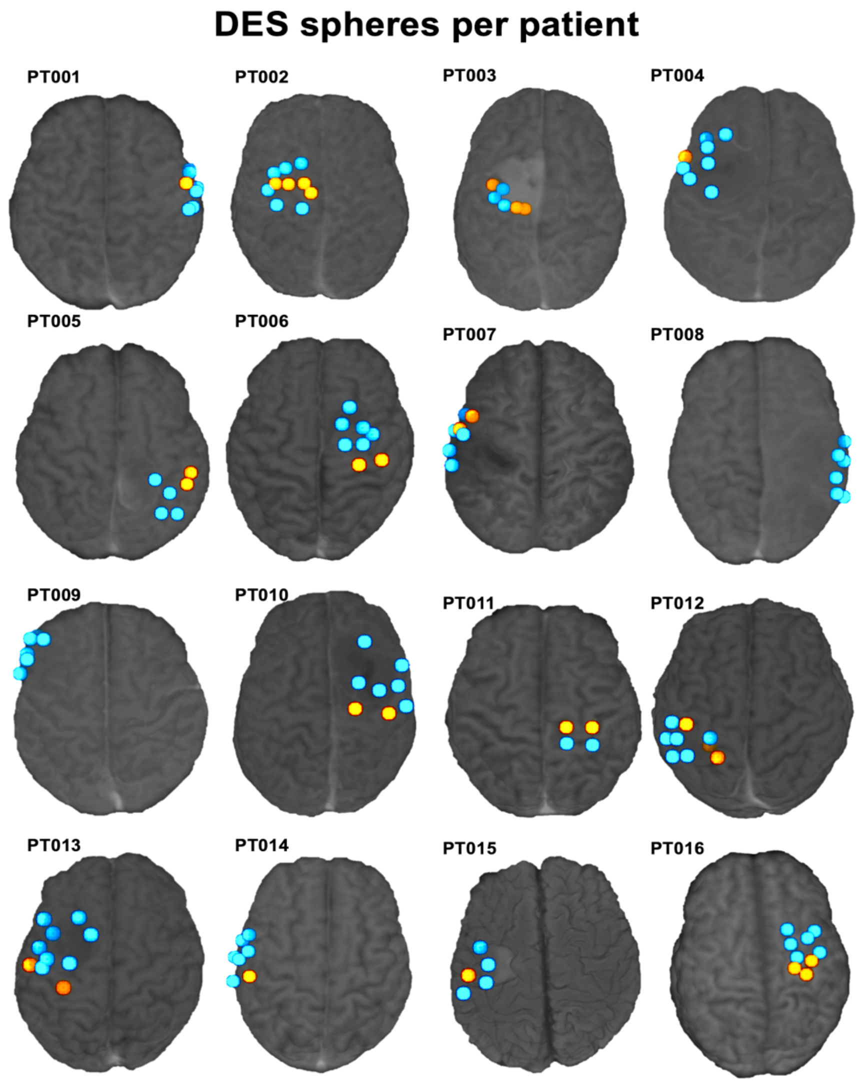

Supplementary figure 3: Semi-transparent 3D renderings of DES spheres for each patient, blue indicates DES coordinates with no response (nDES), and yellow/orange DES coordinates with a response (pDES) overlaid on T1 weighted images in a top-down view

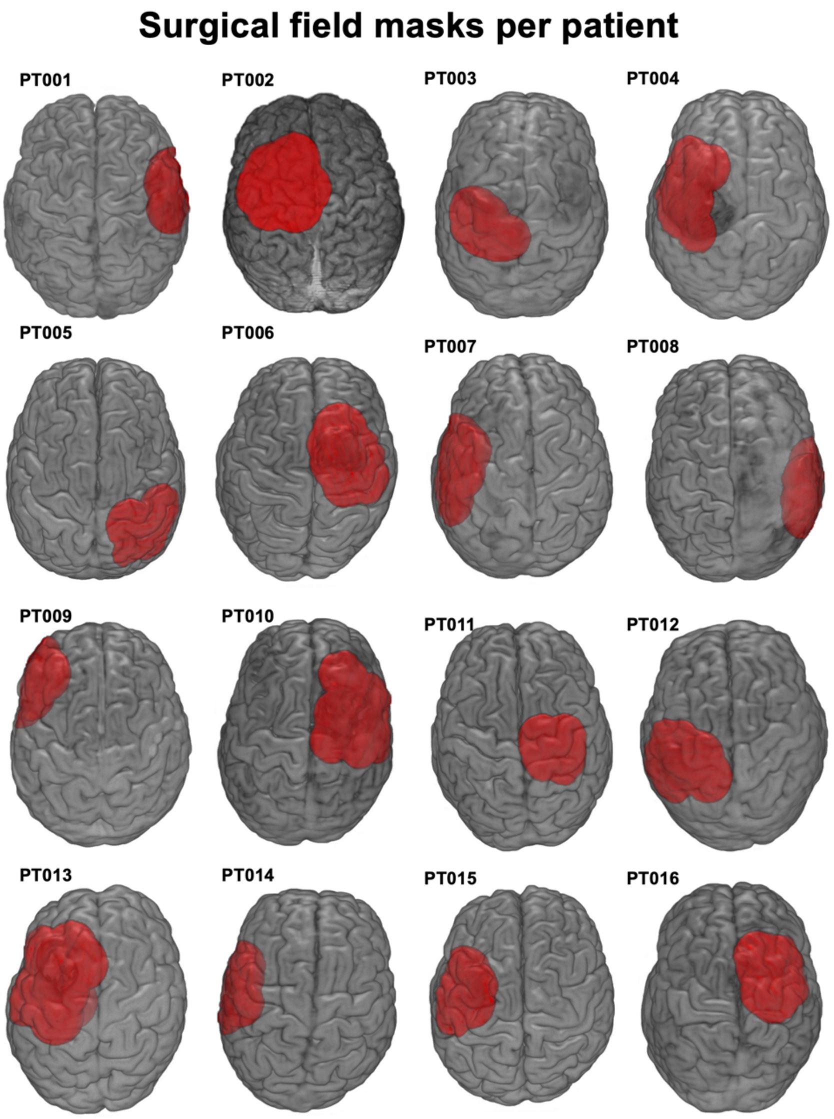

Supplementary figure 4: Semi-transparent 3D renderings of the surgical field masks (red) used in initial distance calculation between DES coordinates and fMRI voxels overlaid on T1 weighted images in a top-down view

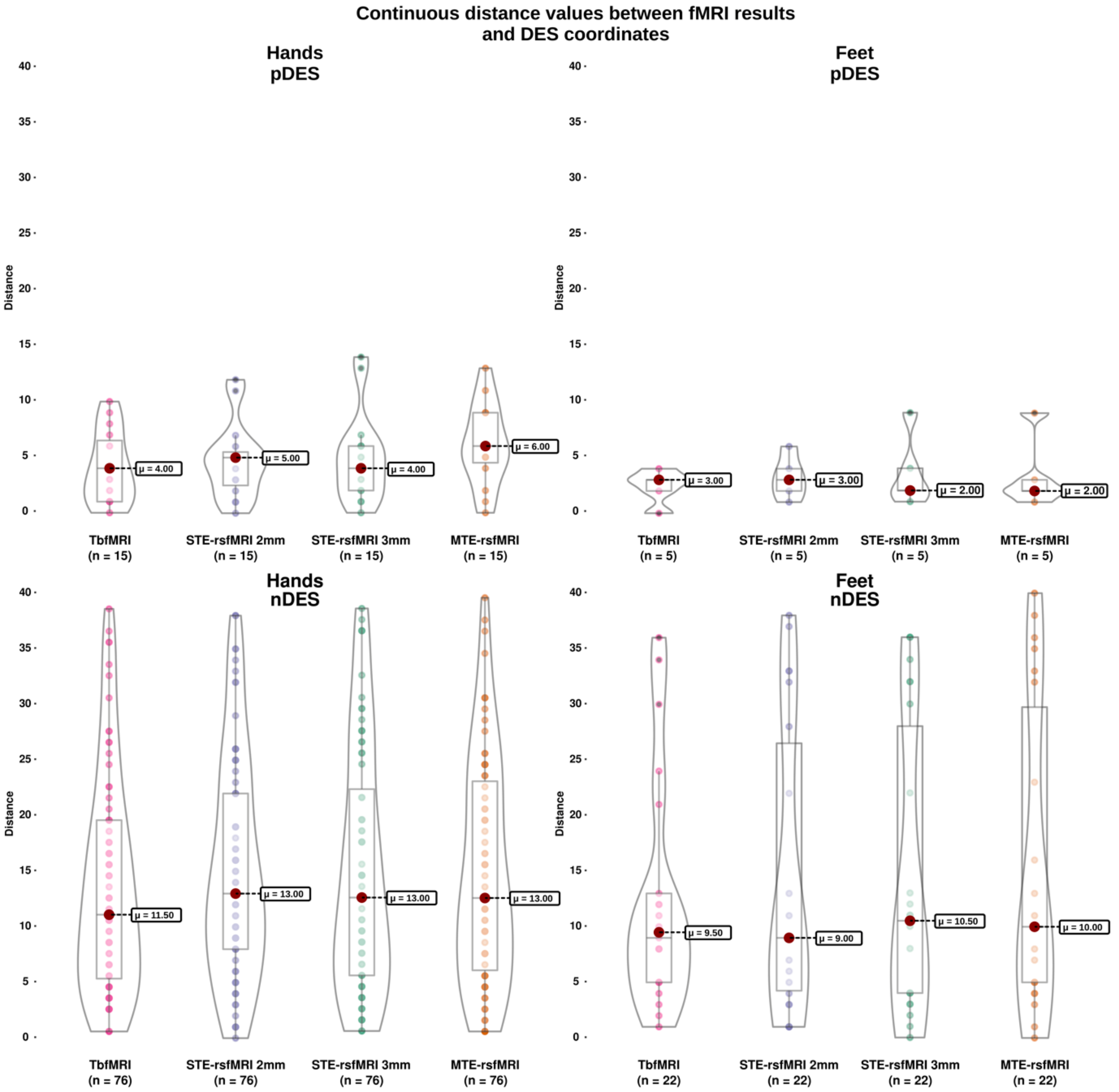

Supplementary figure 5: Distance measures for fMRI and DES responses shown in box and violin plots. Results for the hands are shown on the left, for the feet on the right, for pDES on top, and for nDES on the bottom, tbfMRI = task-based fMRI, sTE-rsfMRI = single-echo resting-state fMRI, mTE-rsfMRI = multi-echo rsfMRI

Supplementary table 1: Patient demographics, pathology results, lesion lobe, side and volume

Coded patient names	Age at scan	Gender	Lesion type	WHO grade	Pathology report	Lesion side and lobe	Lesion volume (ml)
**PT001**	61 – 65	M	Glioma	IV	Glioblastoma	R / Fr-Pa	66.809
**PT002**	6 – 10	F	FCD		Type I	L / Fr	1.200
**PT003**	36 – 40	M	Meningioma	I	Transitional type meningioma	L / Fr	54.079
**PT004**	31 – 35	F	Glioma	II	Oligodendroglioma	L / Fr-Pa	124.775
**PT005**	71 – 75	M	Glioma	IV	Glioblastoma	R / Pa-Oc	32.382
**PT006**	66 – 70	F	Glioma	IV	Glioblastoma	R / Fr	26.779
**PT007**	31 – 35	M	Glioma	IV	Glioblastoma	L / Te-Fr-Pa	88.917
**PT008**	41 – 45	M	Glioma	II	Multifocal astrocytoma	R / Fr-Te-Pa-Oc	123.293
**PT009**	61 – 65	F	Glioma	II	Oligodendroglioma	L / Fr	53.331
**PT010**	31 – 35	M	Glioma	III	Oligodendroglioma	R / Fr	92.154
**PT011**	31 – 35	F	Glioma	II	Oligodendroglioma	L / Pa	22.553
**PT012**	61 – 65	M	Glioma	IV	Glioblastoma	R / Pa	42.517
**PT013**	31 – 35	F	Glioma	III	Oligodendroglioma	L / Fr	46.887
**PT014**	56 – 60	M	Glioma	III	Astrocytoma	L / Fr-Te	40.208
**PT015**	56 – 60	M	Glioma	II	Oligodendroglioma	L / Fr	11.531
**PT016**	11 - 15	M	FCD		Type IIB	R / Fr	4.806

PT = patient, M = male, F = female, FCD = focal cortical dysplasia, WHO = world health organization, R = right, L = left, ml = milliliters, Fr-Pa = frontoparietal, Fr = frontal, Pa-Oc = parieto-occipital, Te-Fr-Pa = Temporo-occipito-parietal, Fr-Te-Pa-Oc = Fronto-temporo-parieto-occipital, Pa = parietal, Fr-Te = fronto-temporal

Supplementary table 2: Direct electrical stimulation (DES) mapping and fMRI details

Coded patient names	Test done	pDES/nDES	DES positive effect	N. of fMRI methods / tasks
**PT001**	Active motor mapping	0/6	none	4 / Hands
**PT002**	Active motor mapping	4/6	Motor response right upper leg, wrist, foot, and hand	4 / Right hand and foot
**PT003**	Active motor mapping	3/3	Motor response right upper leg, foot, and hand	4 / Hands and feet
**PT004**	Active motor mapping	1/7	Motor response right hand, and lower arm	4 / Hands and feet
**PT005**	Active motor mapping	2/4	Interference with finger tapping, and motor response left hand	4 / Hands and feet
**PT006**	Active motor mapping	2/6	Sensory-motor response left leg	2 / Hands and feet
**PT007**	Active motor mapping	0/5	none	4 / Hands
**PT008**	Active motor mapping	0/6	none	4 / Hands
**PT009**	Active motor mapping	0/7	none	4 / Hands
**PT010**	Active motor mapping	1/6	Motor response left hand and foot	2 / Hands
**PT011**	Active motor mapping	1/6	Motor response right hand	4 / Hands
**PT012**	Active motor mapping	2/2	Motor response left foot, leg, hand, and arm	4 / Hands and feet
**PT013**	Active motor mapping	2/8	Motor response right hand	4 / Hands
**PT014**	Active motor mapping	1/6	Motor response right hand	4 / Hands
**PT015**	Active motor mapping	1/4	Motor response right arm	4 / Hands
**PT016**	Active motor mapping	3/6	Motor response left wrist and hand	4 / Hands

PT = patient, DES = direct electrical stimulation, tbMRI = task-based functional magnetic resonance imaging, sTE-rsfMRI = single-echo resting-state fMRI, mTE-rsfMRI = multi-echo rsfMRI, AF = arcuate fasciculus, CST = corticospinal tract, MEP/SSEP = motor and somatosensory evoked potential, B0 = non-diffusion weighted spin-echo EPI volume, 2 = tbfMRI and sTE-rsfMRI 2mm, 4 = tbfMRI, sTE-rsfMRI, and mTE-rsfMRI

Supplementary table 3: Summarized descriptive statistics for similarity measures per fMRI technique (3a) and per fMRI task (3b)

**Supplementary table 3a:** Summarized descriptive statistics for similarity measures per fMRI technique				
**Methods/Measures**	**Metric**	**Max(min)**	**Mean(std)**	**Median(IQR)**
**sTE-rsfMRI 2mm v tbfMRI**	**DSC**	0.516(0.061)	0.235(0.122)	0.203(0.159)
	**JI**	0.348(0.032)	0.138(0.082)	0.113(0.103)
**sTE-rsfMRI 3mm v tbfMRI**	**DSC**	0.526(0.0277)	0.254(0.139)	0.284(0.169)
	**JI**	0.357(0.014)	0.153(0.094)	0.166(0.109)
**mTE-rsfMRI v tbfMRI**	**DSC**	0.450(0.029)	0.239(0.121)	0.260(0.136)
	**JI**	0.290(0.015)	0.140(0.078)	0.150(0.087)
**sTE-rsfMRI 2mm v 3mm**	**DSC**	0.730(0.045)	0.471(0.221)	0.546(0.345)
	**JI**	0.576(0.023)	0.333(0.181)	0.376(0.301)
**sTE-rsfMRI 2mm v mTE-rsfMRI**	**DSC**	0.740(0.044)	0.454(0.219)	0.536(0.341)
	**JI**	0.588(0.022)	0.318(0.180)	0.367(0.288)
**sTE-rsfMRI 3mm v mTE-rsfMRI**	**DSC**	0.744(0.099)	0.525(0.190)	0.557(0.248)
	**JI**	0.871(0.161)	0.655(0.199)	0.728(0.229)
**Supplementary table 3b:** Summarized descriptive statistics for similarity measures per fMRI task				
**Tasks/Measures**	**Metric**	**Max(min)**	**Mean(std)**	**Median(IQR)**
**Hands**	**DSC**	0.744(0.028)	0.367(0.203)	0.333(0.358)
	**JI**	0.851(0.014)	0.291(0.228)	0.200(0.326)
**Feet**	**DSC**	0.740(0.053)	0.338(0.226)	0.269(0.306)
	**JI**	0.871(0.027)	0.271(0.242)	0.164(0.348)
sTE-rsfMRI = singe-echo resting-state fMRI, tbfMRI = task-based fMRI, mTE-rsfMRI = multi-echo rsfMRI, DSC = Dice similarity coefficient, JI = Jaccard index, max = maximum, min = minimum, stdev = standard deviation, IQR = interquartile range				

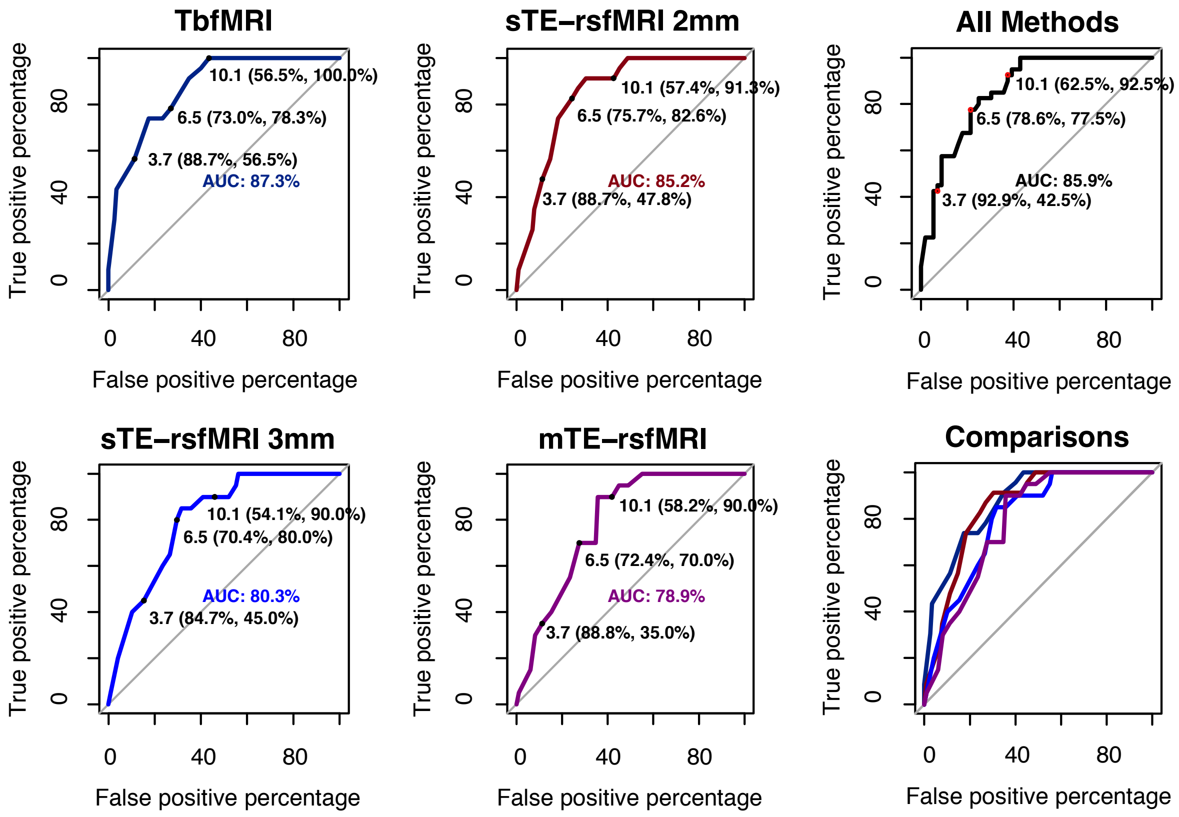

Supplementary figure 6: ROCs of raw distance measures without intra-subject averaging.

Supplementary table 4: Summarized results of DeLong pairwise tests comparing the ROC curves from averaged distance measures

Pairwise comparisons	Estimate1	Estimate2	Statistic	p.value	CI.low	CI.high
**TbfMRI v sTE-rsfMRI 2mm**	92.14	88.21	0.53	0.60	-0.11	0.18
**TbfMRI v sTE-rsfMRI 3mm**	92.14	82.14	1.53	0.13	-0.03	0.23
**TbfMRI v mTE-rsfMRI**	92.14	79.29	1.55	0.12	-0.03	0.29
**sTE-rsfMRI 2mm v 3 mm**	88.21	82.14	1.12	0.26	-0.05	0.17
**sTE-rsfMRI 2mm v mTE-rsfMRI**	88.21	79.29	1.50	0.13	-0.03	0.21
**sTE-rsfMRI 3mm v mTE-rsfMRI**	82.14	79.29	0.57	0.57	-0.07	0.13

ROC = receiver operating characteristic, CI = confidence interval, TbfMRI = task-based fMRI, sTE-rsfMRI = single-echo resting-state fMRI, mTE-rsfMRI = multi-echo resting-state fMRI

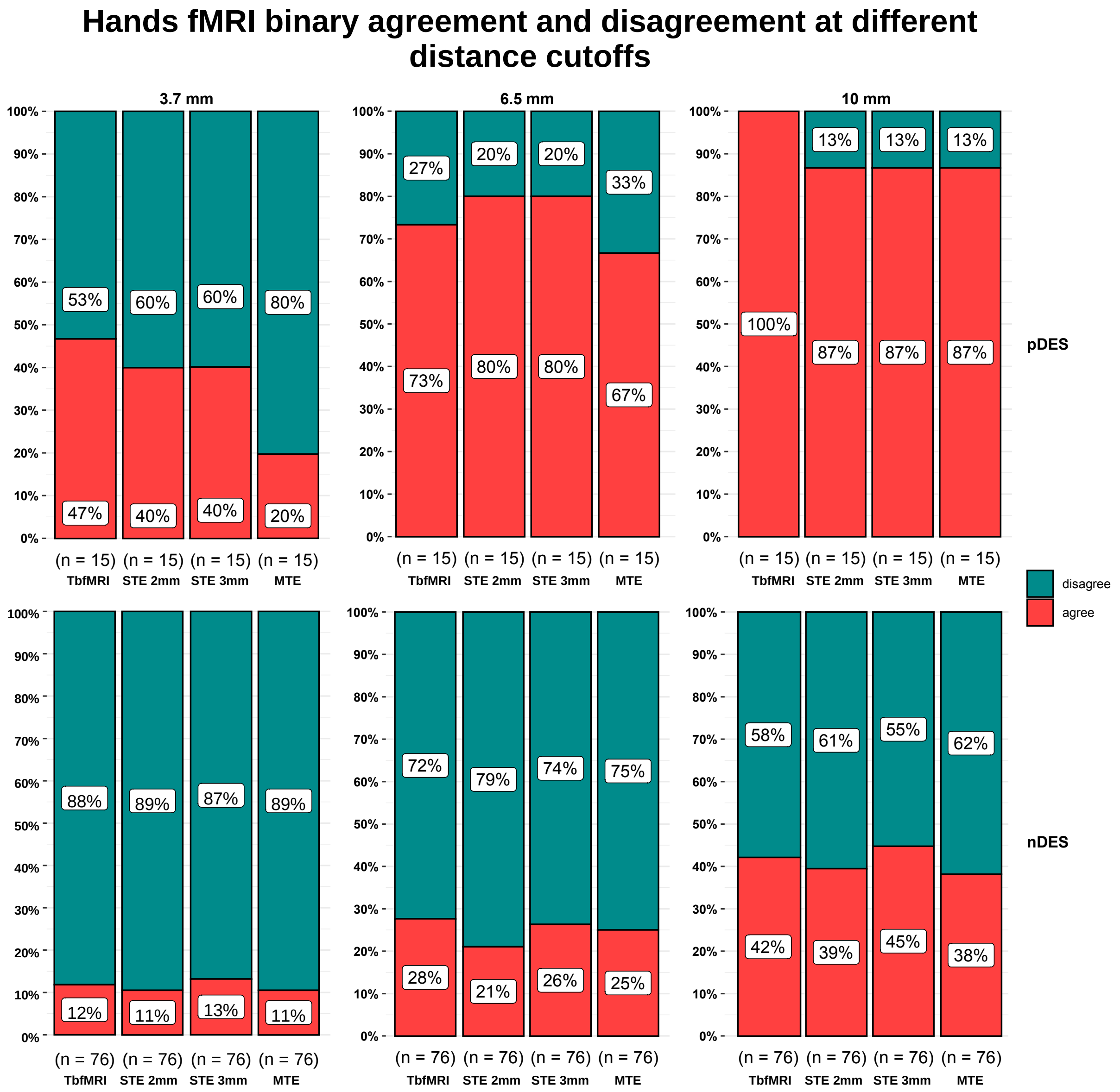

Supplementary figure 7: Stacked column plots depicting hands fMRI percent of agreement versus disagreement with different fMRI methods and DES at ROC-determined distance cutoffs, tbfMRI = task-based fMRI, sTE = single-echo resting-state fMRI, mTE = multi-echo rsfMRI

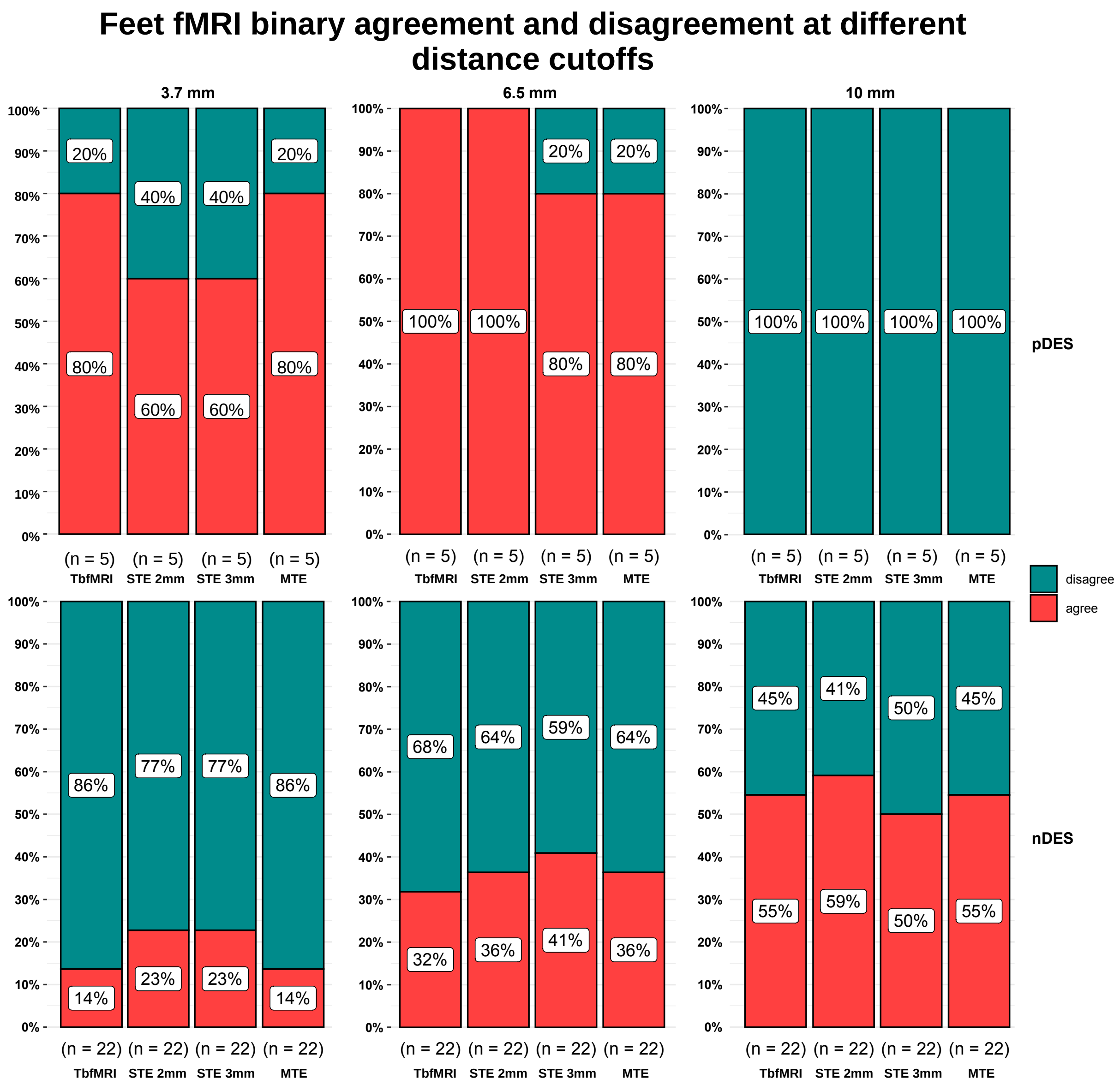

Supplementary Figure 8: Stacked column plots depicting feet fMRI percent of agreement versus disagreement with different fMRI methods and DES at ROC-determined distance cutoffs, tbfMRI = task-based fMRI, sTE-rsfMRI = single-echo resting-state fMRI, mTE-rsfMRI = multi-echo rsfMRI

Two-part linear model details

The first part (A) used a logit-link for binary response (distance=0 vs. distance >0) and a generalized linear mixed model to predict probability of nonoverlap (distance > 0), and the second part (B) used a log-normal linear mixed model for the distance measures (distance >0) between nonoverlapping fMRI-DES coordinate pairs.

The thresholded distance measures were used as the dependent variable and DES response type (positive and negative), fMRI methods (tbfMRI, sTE-rsfMRI 2mm, sTE-rsfMRI 3mm, and mTE-rsfMRI 2mm) were used as predictors in both parts of the model.

For ease of interpretation, we discuss and plot the probability of overlap (distance = 0) for the first part (A), and log distances (B) from the second part are back-transformed to distance in mm.

Results were interpreted in the following context: Predicted probability of overlap (distance < cutoff) with pDES coordinates was analogous to true-positive rate. Predicted probability of overlap with nDES coordinates was analogous to false-positive rate. Predicted distances to non-overlapping (distance > cutoff) nDES coordinates were analogous to true-negative rate and predicted distances to non-overlapping pDES coordinates were analogous to false-negative rate.

In this analysis, missing data was represented and handled by assigning 'NAN' (Not a Number) values. The decision to use 'NAN' ensured that the missing data did not influence the statistical calculations or skew the results. The analysis was then carried out on the remaining dataset, excluding the 'NAN' values.
